# Autophagy of the m^6^A mRNA demethylase FTO is impaired by low-level arsenic exposure to promote tumorigenesis

**DOI:** 10.1038/s41467-021-22469-6

**Published:** 2021-04-12

**Authors:** Yan-Hong Cui, Seungwon Yang, Jiangbo Wei, Christopher R. Shea, Wen Zhong, Fang Wang, Palak Shah, Muhammad G. Kibriya, Xiaolong Cui, Habibul Ahsan, Chuan He, Yu-Ying He

**Affiliations:** 1 Department of Medicine, Section of Dermatology, University of Chicago, Chicago, IL USA; 2 Departments of Chemistry, Department of Biochemistry and Molecular Biology, Institute for Biophysical Dynamics, University of Chicago, Chicago, IL USA; 3 Department of Radiation Oncology, 4th Affiliated Hospital, China Medical University, Shenyang, China; 4 Department of Environmental Health, School of Public Health, China Medical University, Shenyang, China; 5 Institute for Population and Precision Health, Department of Public Health Sciences, The University of Chicago, Chicago, IL USA; 6 Howard Hughes Medical Institute, University of Chicago, Chicago, IL USA; 7 Present Address: Michigan Center for Translational Pathology, University of Michigan, Ann Arbor, MI USA

**Keywords:** Oncogenes, Squamous cell carcinoma

## Abstract

Here we show that FTO as an *N*^6^-methyladenosine (m^6^A) RNA demethylase is degraded by selective autophagy, which is impaired by low-level arsenic exposure to promote tumorigenesis. We found that in arsenic-associated human skin lesions, FTO is upregulated, while m^6^A RNA methylation is downregulated. In keratinocytes, chronic relevant low-level arsenic exposure upregulated FTO, downregulated m^6^A RNA methylation, and induced malignant transformation and tumorigenesis. *FTO* deletion inhibited arsenic-induced tumorigenesis. Moreover, in mice, epidermis-specific *FTO* deletion prevented skin tumorigenesis induced by arsenic and UVB irradiation. Targeting FTO genetically or pharmacologically inhibits the tumorigenicity of arsenic-transformed tumor cells. We identified *NEDD4L* as the m^6^A-modified gene target of FTO. Finally, arsenic stabilizes FTO protein through inhibiting p62-mediated selective autophagy. FTO upregulation can in turn inhibit autophagy, leading to a positive feedback loop to maintain FTO accumulation. Our study reveals FTO-mediated dysregulation of mRNA m^6^A methylation as an epitranscriptomic mechanism to promote arsenic tumorigenicity.

## Abstract (web summary)

RNA m6A demethylase FTO has oncogenic roles in cancers. Here the authors show that chronic low-level exposure of arsenic inhibits autophagic degradation of FTO, leading to FTO stabilisation and reduced m6A RNA methylation in keratinocytes and its subsequent malignant transformation.

## Introduction

Analogous to epigenetic chemical modifications of DNA and histones, RNA modifications regulate RNA fate and gene expression. The most abundant RNA modification is *N*^6^-methyladenosine (m^6^A)^1–4^. m^6^A methylation regulates several aspects of RNA metabolism such as mRNA decay, nuclear processing, translation, transcription, and RNA-protein interactions^1–5^. m^6^A modification plays an important role in development, stem cell homeostasis, and diseases such as cancer^3,6,7^, as well stress response to UV radiation^8^ or heat shock^9^. At the molecular level, RNA m^6^A methylation is installed by writers (methyltransferases), removed by erasers (demethylases), and recognized by readers, a piece of well-coordinated machinery to regulate m^6^A’s dynamics^1–4^. Among these m^6^A effectors, FTO (fat mass and obesity-associated protein) was the first RNA m^6^A demethylase discovered^10^ and has been shown to play oncogenic roles in leukemia^11^, glioblastoma^12^, and melanoma^13^. However, the role of RNA methylation in arsenic-induced skin tumorigenesis remains unclear.

Exposure to inorganic arsenic in contaminated drinking water poses an environmental public health threat for hundreds of millions of people in the US and in the world^14–16^. Arsenic is a natural component of the earth’s crust and is widely distributed throughout the environment. In nature, sources of arsenic exposure include food, air, and water, with the main one being arsenic-contaminated drinking water^17^. Arsenic is a human carcinogen. Chronic exposure to arsenic can cause skin cancer and several internal cancers such as lung, bladder, and kidney cancer^17^. A major target organ of arsenic is the skin. Arsenic-induced skin lesions are an early manifestation of arsenic exposure and toxicity^18^ and are a risk factor for subsequent cancers^19^. Arsenic seems to act in concert with sunlight exposure, smoking, and occupational exposures in increasing the risk of skin lesions^20^. At the molecular level, arsenic has been shown to cause oxidative stress, DNA damage, chromosomal aberration, and epigenetic modifications, including DNA methylation and histone modification^17,21,22^. However, the mechanism by which arsenic causes tumorigenesis is still poorly understood.

In this study, we discovered that chronic low-level arsenic exposure upregulates FTO and downregulates m^6^A RNA methylation in keratinocytes, and induces malignant transformation and tumorigenesis through FTO and m^6^A mRNA methylation. Our study reveals that arsenic inhibits autophagy and autophagic degradation of FTO through suppressing *p62* transcription, leading to FTO stabilization. Furthermore, FTO forms a positive feedback loop with autophagy inhibition to maintain FTO accumulation in arsenic tumorigenesis. Taken together, these results demonstrate that m^6^A RNA methylation acts as an epitranscriptomic mechanism for arsenic damage response and tumorigenesis.

## Results

### FTO is upregulated, while m^6^A is downregulated, by arsenic in human keratinocytes and in epidermal keratinocytes in human skin

**Fig. 1:**
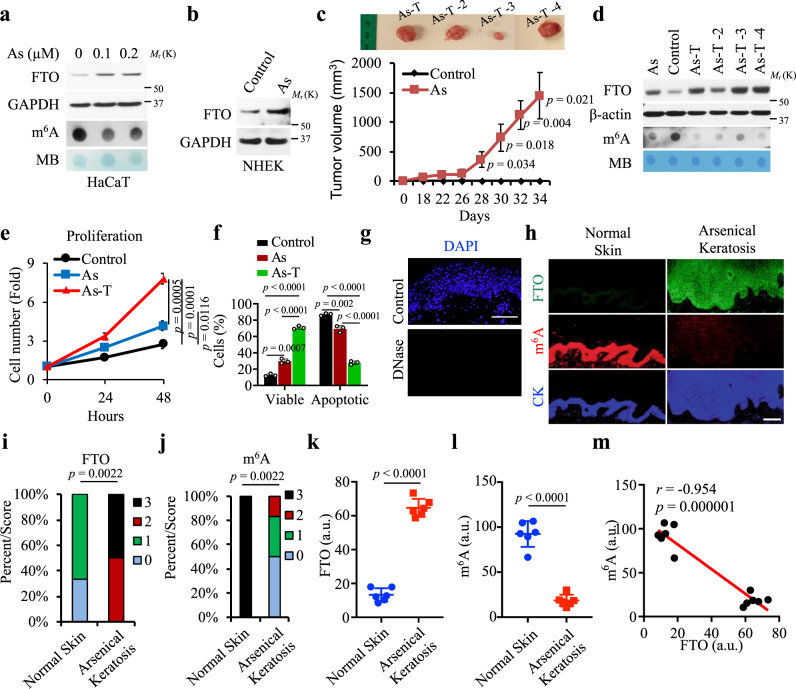
FTO is upregulated, while m^6^A is downregulated by arsenic in human skin and keratinocytes. **a** Immunoblot analysis of FTO and m^6^A dot blot assay in HaCaT cells treated with arsenic (As) at different doses for 72 h. Methylene blue (MB) staining was used as a loading control. **b** Immunoblot analysis of FTO in NHEK cells. **c** Tumor images (upper) and volume (lower) following subcutaneous injection of control and arsenic-treated cells (As) (5 × 10^6^) in nude mice (*n* = 4). **d** Immunoblot analysis of FTO and m^6^A dot blot assay in control, As, and the four As-T cells. Methylene blue (MB) staining was used as a loading control. **e** Cell proliferation assay in control, As, and As-T cells (*n* = 3). **f** Cell viability assay in cells as in **e** but placed in suspension for 72 h using flow cytometric analysis following Annexin V/propidium iodide staining (*n* = 3). **g** Nuclei/DNA are stained with DAPI in blue in normal human skin treated with or without DNase I (20 U). Scale bar, 100 μm. **h** Immunofluorescence staining of FTO (green), m^6^A (red), and keratin (blue) in normal human skin (*n* = 6) and arsenical keratoses (*n* = 6). Scale bar, 100 μm. **i**, **j** Percentage of samples (in stacked column format) for each score of FTO from **h**. Mann–Whitney *U* test. **k**, **l** Quantification of FTO and m^6^A levels using Image J (1.53e) from **h**. **m** Negative correlation of m^6^A levels with FTO levels by Spearman’s test from **h**. All data were performed on *n* ≥ 3 biologically independent samples. Error bars are shown as mean ± S.D. (**e**, **f**, **k**, **l**) or mean ± S.E. **c** *p*-values by two-tailed unpaired *t*-tests (**c**, **e**, **f**, **k**, **l**). Mann–Whitney *U* test (**i**, **j**). Correlation coefficient *r* and *p*-value are indicated from Spearman’s Correlation Rank test (**m**). a.u.: arbitrary units (**k**–**m**).

To determine whether m^6^A RNA methylation and its regulators play a role in arsenic-induced tumorigenicity, we first treated HaCaT cells, a human keratinocyte cell line, continuously with a relevant low level (100 nM) of inorganic arsenite for 28 weeks to generate HaCaT cells with chronic arsenic damage (As cells)^23,24^ (Supplementary Fig. 1a). Arsenic substantially upregulated FTO and downregulated m^6^A, while it had moderate or no effect on other m^6^A regulators, including the m^6^A methyltransferase proteins METTL14 and METTL3 and the m^6^A demethylase ALKBH5 (Supplementary Fig. 1b). We found that treatment with a low level of arsenic for up to 72 h upregulated FTO protein and downregulated m^6^A in HaCaT cells (Fig. 1a and Supplementary Fig. 1c). The effect of arsenic on FTO seemed to be dose-dependent, since only low-level arsenic upregulated FTO, whereas high-level arsenic downregulated FTO (Supplementary Fig. 1d). Similarly, FTO upregulation was also detected in NHEK cells (normal human epidermal keratinocytes) (Fig. 1b).

As-treated cells developed tumors in all four mice, while control cells did not (Fig. 1c and Supplementary Fig. 1e). We isolated the tumorigenic cells (As-T, Arsenic-Tumorigenic cells) from one tumor in mice (#1) as well as other tumors formed by the As-treated cells (As cells) in mice (Fig. 1c and Supplementary Fig. 1a). In addition to As cells, FTO was also upregulated in the four As-T cells, while the m^6^A level was downregulated in As and four As-T cells as compared with control cells (Fig. 1d). In addition, as compared with control cells, As and As-T cells showed increased proliferation (Fig. 1e) and survival (Fig. 1f, and Supplementary Fig. 1f). However, in contrast to arsenic, cadmium, another carcinogenic heavy metal present in the air, water, soil, sediment^25^, had no effect on FTO in HaCaT cells (Supplementary Fig. 1g), suggesting FTO as a target unique for arsenic exposure. Future investigation is needed to determine whether cadmium and other heavy metals affect FTO or other m^6^A regulators in keratinocytes and other cell types.

To determine the role of FTO and m^6^A in arsenic-induced tumorigenesis, we next assessed whether the levels of m^6^A and FTO are altered in arsenic-induced premalignant human skin lesions, arsenical keratoses, as compared with normal human skin. In humans, it is known that chronic arsenic exposure causes arsenical keratoses, which can persist indefinitely and may develop into invasive skin cancers such as squamous cell carcinoma^26^. Normal human skin samples were obtained from the archives in the tissue bank of the Section of Dermatology (Department of Medicine, University of Chicago). The arsenical keratoses were obtained from the clinical follow-up for arsenic-exposed cohorts that evaluate the health effects of exposure, including skin cancer, in Bangladesh. To remove the signal from DNA, we treated all samples with DNase I^8^ (Fig. 1g). As compared with normal human skin, we found that FTO is upregulated and the m^6^A level is downregulated in arsenical keratoses (Fig. 1h–l). In addition, we found that the m^6^A level is negatively correlated with the FTO level (Fig. 1m). These findings suggest that FTO acts as a protumorigenic factor in arsenic-induced skin lesions.

### FTO is required for arsenic-induced tumorigenicity

**Fig. 2:**
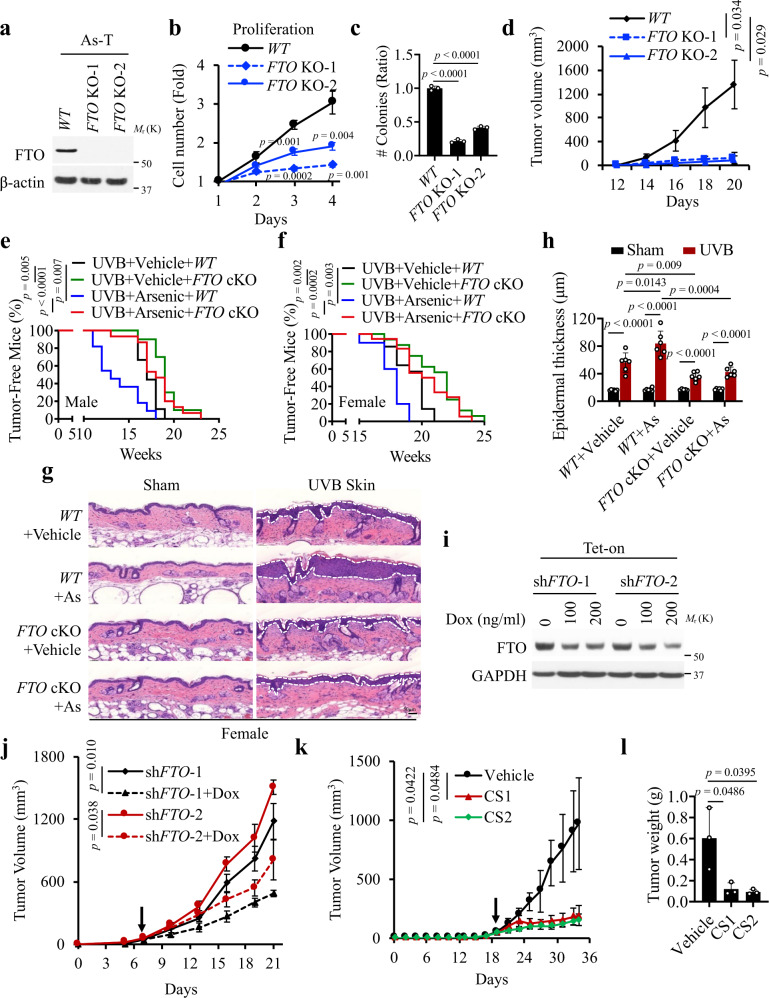
FTO is required for arsenic-induced malignant transformation and tumorigenicity. **a** Immunoblot analysis confirming *FTO* knockout (KO) in As-T cells using two independent sgRNAs using CRISPR. **b** Cell proliferation assay in As-T with or without *FTO* deletion (*n* = 3). **c** Soft Agar assay of cells as in **a** (*n* = 3). **d** Tumor volume of As-T cells with or without *FTO* deletion in NSG mice (*n* = 4). **e**, **f** Kaplan–Meier curve of tumorigenesis in UVB-irradiated male (**e**) and female (**f**) mice with wild-type *FTO* (*WT*; *FTO* ^*flox/lox*^) or skin-specific *FTO* conditional knockout (*FTO* cKO; *K14Cre*; *Fto*^*flox/flox*^) with or without arsenic exposure. UVB + Vehicle + WT (Male, *n* = 9; Female, *n* = 14); UVB + Vehicle+*FTO* cKO (Male, *n* = 10; Female, *n* = 16); UVB + Arsenic+*WT* (Male, *n* = 11; Female, *n* = 10); UVB + Arsenic+*FTO* cKO (Male, n = 15; Female *n* = 18). **g**, **h** Quantification of an epidermal thickness (**h**) from histological analysis (HE staining) (**g**) in Female mice. Sham group: *WT* + Vehicle (*n* = 6); *WT* + As (*n* = 6); *FTO* cKO + Vehicle (*n* = 5); *FTO* cKO+As (*n* = 6); groups treated with UVB (*n* = 6). **i** Immunoblot analysis of FTO and GAPDH in As-T cells stably infected with doxycycline-inducible shRNA 1 and 2 targeting *FTO* (Tet-on sh*FTO*-1 and sh*FTO*-2). **j** Tumor growth in nude mice treated with or without doxycycline following inoculating cells as in **i**. Tet-on sh*FTO*-1(*n* = 4), and sh*FTO*-2 (*n* = 3). **k**, **l** Tumor growth (**k**) and weight (**l**) in nude mice bearing As-T cells upon treatment with vehicle, CS1 (5 mg/kg), or CS2 (5 mg/kg). All data were performed on *n* ≥ 3 biologically independent samples. Error bars are shown as mean ± S.D. (**b**, **c**, **h**, **k**, **l**) or mean ± S.E. (**d**, **j**). *p*-values by two-tailed unpaired *t*-tests (**b**, **c**, **d**, **h**, **j**, **k**, **l**). Log-rank (Mantel-Cox) tests are indicated (**e**, **f**).

To determine the functional significance of FTO upregulation in arsenic-induced tumorigenicity, we assessed the consequence of FTO knockout (KO) in As-T cells using CRISPR (Fig. 2a). *FTO* deletion decreased cell growth (Fig. 2b), anchorage-independent growth (Fig. 2c), clonogenic growth (Supplementary Fig. 2a), 3D cell growth (Supplementary Fig. 2b), and survival when cells were placed in suspension (Supplementary Fig. 2c). Notably, *FTO* deletion partially reversed the effect of arsenic on cell survival (Supplementary Fig. 2c). It is possible that FTO and other pathways affected by arsenic also promote cell survival. Nevertheless, *FTO* deletion drastically inhibited tumor growth of As-T cells in mice (Fig. 2d and Supplementary Fig. 2d). Furthermore, knockdown of *FTO* using siRNA also decreased cell proliferation in As cells (Supplementary Fig. 2e). While overexpression of FTO slightly increased cell proliferation in HaCaT cells (Supplementary Fig. 2f), it did not induce tumor formation in NSG mice (Supplementary Fig. 2g), indicating that FTO overexpression alone is not sufficient to induce tumorigenesis. It is likely that FTO upregulation is critical for arsenic tumorigenesis, while it seems that both FTO upregulation and other molecular alterations induced by arsenic are required for malignant transformation and tumorigenesis.

Next, we used the arsenic-UVB skin co-carcinogenesis mouse model to determine the role of FTO in arsenic-induced skin tumorigenesis. We first created mice with epidermis-specific *FTO* deletion (*K14Cre*;*Fto* ^*flox/flox*^). Then we treated the mice with arsenic, UVB irradiation, or both, as reported previously^27^. We found that arsenic accelerates skin tumorigenesis, consistent with the previous report^27^. Epidermis-specific *FTO* deletion inhibited skin tumorigenesis induced by UVB irradiation alone or the combination of UVB irradiation and arsenic, with a greater effect on the latter (Fig. 2e, f). In addition, skin-specific *FTO* deletion reduced epidermal hyperplasia induced by UVB irradiation alone or the combination of UVB irradiation and arsenic (Fig. 2g, h). Histological analysis showed that the tumors developed in these groups are either papillomas or squamous cell carcinomas (SCC) (representative images shown in Supplementary Fig. 2h).

To determine the therapeutic potential by targeting FTO in As-T cells, we first tested the effect of inducible *FTO* knockdown using a doxycycline-inducible shRNA knockdown system. Inducible *FTO* knockdown (Fig. 2i) decreased cell growth (Supplementary Fig. 2i), tumor growth (Fig. 2j), and tumor weight (Supplementary Fig. 2j). To determine the translational potential of targeting FTO, we tested the effect of the FTO inhibitors CS1 and CS2 identified recently^28^. CS1 and CS2 increased the m^6^A level (Supplementary Fig. 2k), while it decreased cell growth (Supplementary Fig. 2l), tumor growth (Fig. 2k), and tumor weight (Fig. 2l), supporting the anti-tumor effects of these FTO inhibitors in As-T cells. Overall, these findings demonstrate that FTO is required for arsenic-induced malignant transformation and tumorigenesis and that targeting FTO genetically or pharmacologically inhibits the tumorigenicity of arsenic-treated cells.

### m^6^A seq and RNA seq analyses identify NEDD4L as the critical target for arsenic-induced FTO upregulation

**Fig. 3:**
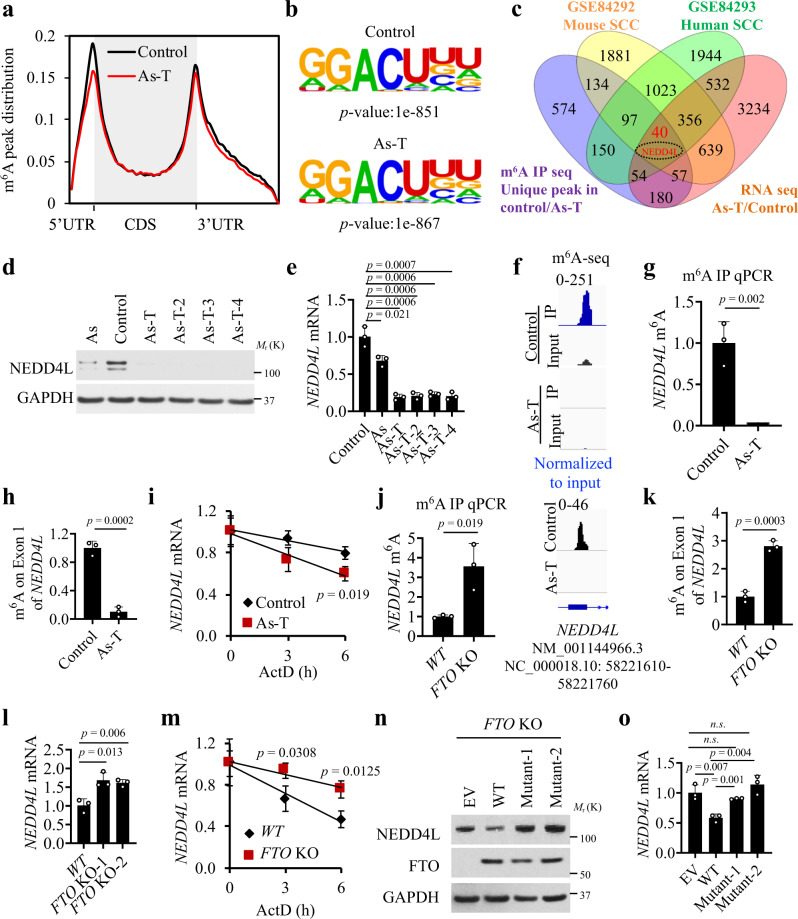
Identification of the potential target of arsenic-induced FTO upregulation via transcriptome-wide m^6^A-seq and RNA-seq analyses. **a** Distribution of m^6^A peaks across the length of mRNA in control and As-T cells. **b** Top consensus m^6^A motif identified by HOMER with m^6^A peaks in control and As-T cells. **c** Venn diagram showing the overlap between genes with altered expression or m^6^A enrichment in four datasets: m^6^A seq (unique m^6^A peak in control/As-T), RNA seq (As-T/control), RNA seq [Mouse cuSCC/chronically irradiated skin (CHR)], and RNA seq [human cuSCC/normal skin (NS)]. *NEDD4L* is found in all groups. **d** Immunoblot analysis of FTO and GAPDH in control, As, and four As-T cells. **e** qPCR analysis of *NEDD4L* mRNA levels in **d** (*n* = 3). **f** Distribution of m^6^A peaks in “Exon 1 (NC_000018.10:58221610-58221760)” of the *NEDD4L* transcript in control and As-T cells. **g** m^6^A IP qPCR analysis of m^6^A enrichment across the *NEDD4L* transcript in control and As-T cells (*n* = 3). **h** m^6^A IP qPCR analysis of m^6^A enrichment in the differentially methylated region of the *NEDD4L* transcript (NC_000018.10:58221610-58221760) in control and As-T cells (*n* = 3). **i** qPCR analysis of *NEDD4L* mRNA stability following treatment with actinomycin. D (ActD, 2 μM) in control and As-T cells (*n* = 3). **j** m^6^A IP qPCR analysis of m^6^A enrichment across the *NEDD4L* transcript in As-T cells with or without *FTO* deletion (*n* = 3). **k** m^6^A IP qPCR analysis of m^6^A enrichment in the differentially methylated region of the *NEDD4L* transcript (NC_000018.10:58221610-58221760) in wild-type (WT) and FTO KO cells (*n* = 3). **l** qPCR analysis of *NEDD4L* mRNA level in cells with or without *FTO* deletion (*n* = 3). **m** qPCR analysis of *NEDD4L* mRNA stability following treatment with ActD in As-T cells with or without *FTO* deletion (*n* = 3). **n** Immunoblot analysis of NEDD4L and FTO in FTO-KO cells transfected with wild-type (WT) or demethylase-inactive mutant FTO (Mutant 1, H231A/D233A; Mutant 2, R316Q/R322Q). **o** qPCR analysis of *NEDD4L* mRNA levels in cells as in **n** (*n* = 3). All data were performed on *n* = 3 biologically independent samples. Error bars are shown as mean ± S.D. **e**, **g**–**m**, **o** *p*-values by two-tailed unpaired *t*-tests are indicated (**e**, **g**–**m**, **o**).

To determine the potential m^6^A targets of arsenic exposure, we used m^6^A-seq coupled with RNA-seq to determine the potential m^6^A demethylation gene targets across the whole transcriptome for arsenic. As compared with control cells, As-T cells showed decreased m^6^A enrichment in 5’UTR and 3’UTR (Fig. 3a) and altered total peak distribution (Supplementary Fig. 3a). As-T cells also showed a decreased number of m^6^A genes compared with control cells (Supplementary Fig. 3b). Sequence analysis of m^6^A peaks showed the previously identified m^6^A target sites (GGACU)^29,30^ (Fig. 3b).

Next, we used the following criteria and analyses and identified *NEDD4L* (neural precursor cell-expressed developmentally downregulated 4 like) as a potential m^6^A target of arsenic, m^6^A modification, and FTO: (1) decreased m^6^A enrichment, (2) altered mRNA level, (3) potential role in cancer, and (4) altered expression in both human and mouse SCC. We compared the genes with altered expression and m^6^A enrichment in our m^6^A seq and RNA seq datasets from control and As-T cells, with genes with altered expression from human and mouse skin cancers (squamous cell carcinoma, SCC) (GSE84292 and GSE84293) generated from the previous studies^31^. There were 40 genes altered among the four groups, including 10 downregulated genes (*GSN*, *LPIN1*, *ME1*, *NCOA7*, *NEDD4L*, *PDLIM4*, *TRPV4*, *CLDN1*, *CRIP2*, *GAS6*) and 3 upregulated genes (*GINS4*, *RAD54L*, *SLC16A1*) that shared changes in As-T, mouse and human cuSCCs (Fig. 3c, and Supplementary Fig. 3c). Among all these genes, we elected to initially focus on *NEDD4L*, as it is found to be downregulated in several cancers^32–35^, suggesting a tumor-suppressive role for NEDD4L.

As and four As-T cells showed decreased mRNA and protein levels of NEDD4L as compared with control cells (Fig. 3d, e). m^6^A-seq analysis showed that As-T cells exhibited decreased m^6^A levels in the exon 1 (NC_000018.10:58221610-58221760) region of the *NEDD4L* isoform (id = NM_001144966.3) (Fig. 3f). Consistently, m^6^A IP qPCR analysis showed that in As-T cells the m^6^A level for the *NEDD4L* full-length transcript was reduced as compared with control cells (Fig. 3g), consistent with elevated FTO in these cells (Fig. 1d). To determine the m^6^A site at the *NEDD4L* transcript regulated by FTO, we performed the m^6^A IP qPCR analysis of m^6^A enrichment in the differentially methylated region of the NEDD4L transcript in control and As-T cells. As-T showed decreased m^6^A enrichment in the exon 1 (NC_000018.10:58221610-58221760) region of *NEDD4L* as compared with control cells (Fig. 3h). Furthermore, *NEDD4L* mRNA stability was decreased in As-T compared with control cells (Fig. 3i), suggesting that arsenic regulates *NEDD4L* RNA metabolism and decay. *FTO* deletion increased the m^6^A level and abundance of the *NEDD4L* transcript (Fig. 3j). m^6^A IP qPCR analysis of m^6^A enrichment showed that *FTO* deletion increased the m^6^A level in the exon 1 region of *NEDD4L* in As-T cells (Fig. 3k). Future investigation is needed to determine the importance of other potential m^6^A sites in the *NEDD4L* genes in arsenic tumorigenesis. Moreover, we found that *FTO* deletion increased the *NEDD4L* mRNA and protein levels and mRNA stability (Fig. 3l, m, and Supplementary Fig. 3d), suggesting that increased m^6^A enrichment on the *NEDD4L* transcript elevates its stability instead of leading to transcript decay^36,37^.

To determine whether FTO regulates *NEDD4L* mRNA m^6^A modification and expression through its demethylase activity, we tested the effect of the FTO demethylase-inactive mutants. In cells with *FTO* deletion, wild-type (WT) FTO decreased *NEDD4L* mRNA and protein levels, while FTO demethylase-inactive mutants had no effect (Fig. 3n, o). We also showed that the FTO inhibitors CS1 and CS2 increased the protein and mRNA levels of *NEDD4L* (Supplementary Fig. 3e, f), while it had no effect on the protein or mRNA levels of FTO, consistent with the previous studies^28^. These data strongly indicate that FTO upregulation by arsenic decreases the *NEDD4L* mRNA stability through its RNA demethylase activity.

### Identification of NEDD4L as the functional target of FTO

**Fig. 4:**
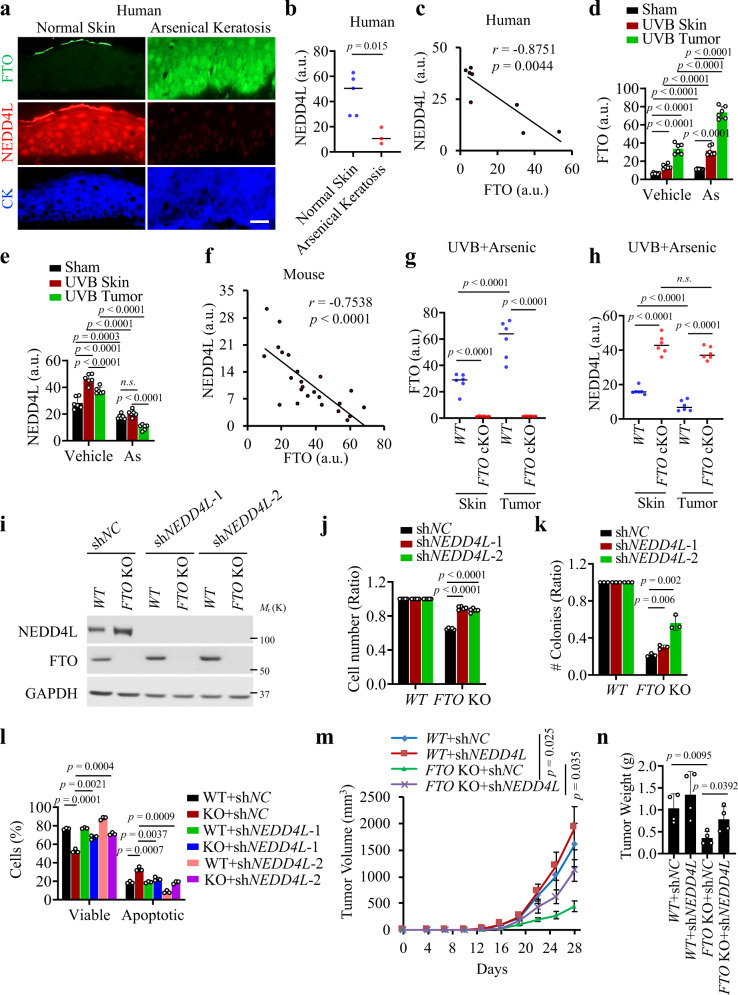
NEDD4L is a critical downstream target of FTO. **a** Immunofluorescence staining of FTO (green), NEDD4L (red), and keratin (blue) in normal humans skin (*n* = 5) and arsenical keratoses (*n* = 3). Scale bar, 20 μm. **b** Quantification of NEDD4L using Image J in **a** normal human skin (*n* = 5) and arsenical keratoses (*n* = 3). **c** Negative correlation of NEDD4L levels with FTO levels by Spearman’s test in **a**. **d** Quantification of FTO using Image J within skin area with sham, UVB non-tumor skin or tumor in wild-type (WT) female mouse with or without arsenic exposure (*n* = 6) related in Supplementary Fig. 4a. **e** Quantification of NEDD4L in non-tumor skin or tumors from female WT mice with or without arsenic or UVB irradiation (*n* = 6). Representative images are shown in Supplementary Fig. 4a. **f** Negative correlation of NEDD4L levels with FTO levels by Spearman’s test in skin and tumor samples from WT mice treated with UVB irradiation in combination with vehicle or arsenic. **g**, **h** Quantification of the levels of FTO (**g**) (*n* = 6) and NEDD4L (**h**) (*n* = 6) in non-tumor skin or tumors from WT and FTO cKO female mice treated with arsenic and UVB irradiation (*n* = 6) from Supplementary Fig. 4b. **i** Immunoblot analysis to confirm *NEDD4L* knockdown in As-T cells with or without *FTO* deletion. **j** Cell proliferation assay in cells as in **i** (*n* = 3). **k** Soft agar assay of cells as in **i** (*n* = 3). **l** Apoptosis assay in cells as in **i** (*n* = 3). **m**, **n** Tumor volume (**m**) and weight (**n**) following subcutaneous injection of cells as in **i** into NSG mice (*n* = 4). All data were performed on *n* ≥ 3 biologically independent samples. Error bars are shown as mean ± S.D. (**b**, **d**, **e**, **g**, **h**, **j**, **k**, **l**, **n**) or mean ± S.E. (**m**). *p*-values by two-tailed unpaired *t*-tests (**b**, **d**, **e**, **g**, **h**, **j**, **k**, **l**, **m**, **n**). Correlation coefficient *r* and *p*-value from Spearman’s Correlation Rank test (**c**, **f**). a.u.: arbitrary units (**b**–**h**).

Next, we investigated whether NEDD4L is responsible for FTO’s function in arsenic tumorigenicity. First, we assessed whether NEDD4L is negatively associated with FTO in arsenical keratoses, as compared with normal human skin. We found that FTO is upregulated, while NEDD4L is downregulated in arsenical keratoses as compared with normal skin (Fig. 4a, b). NEDD4L levels were negatively correlated with FTO levels (Fig. 4c).

Second, we analyzed the alteration of NEDD4L in arsenic-UVB-treated skin and tumors in mice and the effect of *FTO* deletion. We found FTO is upregulated by UVB alone, arsenic alone, or the combination of both (Fig. 4d and Supplementary Fig. 4a). Moreover, while NEDD4L was upregulated by UVB irradiation, it was downregulated by arsenic (Fig. 4e). Other mechanisms may mediate UVB-induced NEDD4L upregulation, which remains to be investigated in detail. Nevertheless, NEDD4L levels were negatively correlated with FTO levels (Fig. 4f). Furthermore, *FTO* deletion increased NEDD4L levels in skin and tumors from mice treated with both UVB irradiation and arsenic (Fig. 4g, h, and Supplementary Fig. 4b).

Third, we knocked down *NEDD4L* and observed that it partially reversed the effect of *FTO* deletion on cell proliferation, anchorage-independent growth, and cell survival when placed in suspension (Fig. 4i-l). Consistently, *NEDD4L* knockdown also partially reversed the effect of *FTO* deletion on tumor growth in vivo (Fig. 4m, n). These data indicate that NEDD4L is an important target of FTO, and other FTO targets may also play an important role in FTO’s function.

Next, we investigated the potential target of NEDD4L. NEDD4L is shown to act as a ubiquitin ligase controlling several pathways, including the WNT signaling by targeting Dvl2^38^. As WNT signaling is one critical pathway in skin cancer^39^, we elected to test the role of the Wnt/β-catenin pathway in NEDD4L’s function. Using TOP Flash luciferase assay, we found that WNT signaling is increased in As-T cells as compared with control cells (Supplementary Fig. 4d). We observed that Dvl2, as well as β-catenin, a downstream target of WNT/Dvl2^40^, is upregulated in As-T cells as compared with control cells (Supplementary Fig. 4e). *FTO* deletion decreased the levels of Dvl2 and β-catenin and WNT activity, which was prevented by *NEDD4L* knockdown (Supplementary Fig. 4e-f). Future investigation is needed to elucidate the role of other pathways, including the TGF-β^41^ and PI3K/AKT^42^ pathways, in the function of NEDD4L in skin cancer. These results demonstrate that NEDD4L is a critical downstream target of FTO responsible for its function in arsenic-induced malignant transformation.

### FTO regulates *NEDD4L* mRNA stability through m^6^A and IGF2BPs

**Fig. 5:**
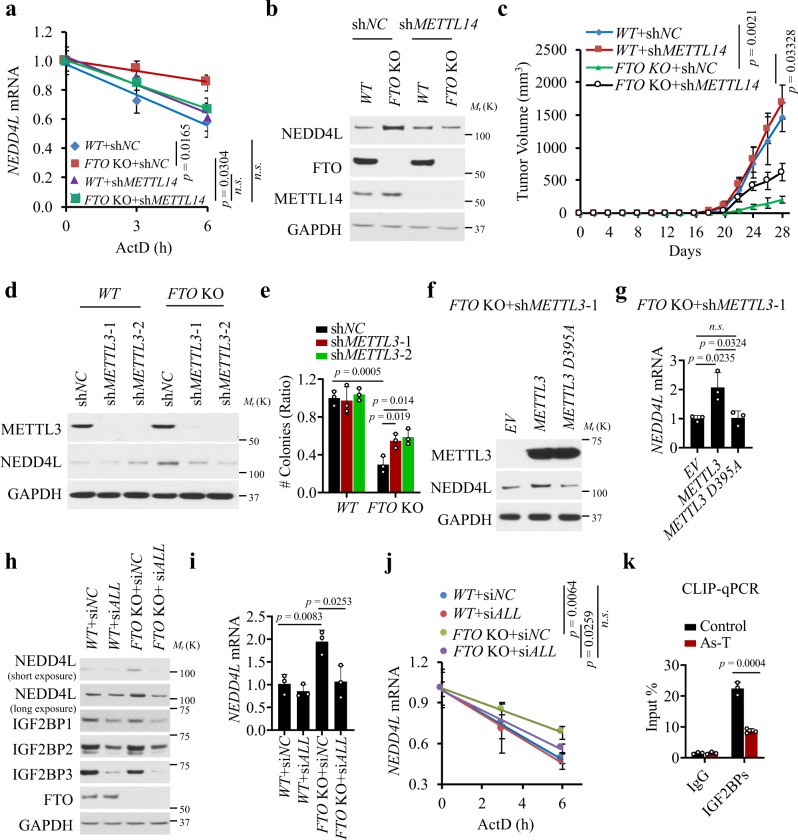
FTO regulates NEDD4L and arsenic tumorigenicity through m^6^A. **a** qPCR analysis of *NEDD4L* mRNA stability in As-T cells with or without *FTO* deletion and/or *METTL14* knockdown (*n* = 3). **b** Immunoblot analysis of NEDD4L, METTL14, and FTO in cells as in **a**. **c** Tumor volume as in **a** following subcutaneous injection of cells as in **a** into NSG mice (*n* = 4). **d** Immunoblot analysis of NEDD4L and METTL3 in As-T cells with or without *FTO* knockout and/or *METTL3* knockdown. **e** Soft agar assay of cells as in **d** (*n* = 3). **f** Immunoblot analysis of NEDD4L and METTL3 in cells with *FTO* deletion and *METTL3* knockdown transfected with wild-type (WT) *METTL3* or demethylase-inactive mutant *METTL3 D395A*. **g** qPCR analysis of *NEDD4L* mRNA level in cells as in **f** (*n* = 3). **h** Immunoblot analysis of NEDD4L, IGF2BP1, IGF2BP2, IGF2BP3, and FTO in As-T cells with or without *FTO* deletion in combination with transfection with siRNA targeting negative control (si*NC*) or all three *IGF2BP1-3* (si*ALL*). **i** qPCR analysis of *NEDD4L* mRNA level in cells as in **h** (*n* = 3). **j** qPCR analysis of *NEDD4L* mRNA stability in cells as in **h** (*n* = 3). **k** CLIP-qPCR showing the binding of IGF2BPs to the *NEDD4L* transcript in control and As-T cells (*n* = 3). All data were performed on *n* ≥ 3 biologically independent samples. Error bars are shown as mean ± S.D. (**a**, **e**, **g**, **i**–**k**) or mean ± S.E. **c** *p*-values of all data by two-tailed unpaired *t*-test are indicated.

To determine whether FTO regulates NEDD4L through m^6^A, we assessed the effect of manipulating the m^6^A RNA methyltransferase factors METTL14 and METTL3^43^. *METTL14* knockdown at least partially reversed the effect of *FTO* deletion on *NEDD4L* mRNA stability, mRNA level, and protein level (Fig. 5a, b, and Supplementary Fig. 5a). Furthermore, *METTL14* knockdown partially reversed the effect of *FTO* deletion on tumor growth (Fig. 5c and Supplementary Fig. 5b). Similarly, *METTL3* knockdown also partially reversed the effect of *FTO* deletion on cell growth (Fig. 5d and Supplementary Fig. 5c), and anchorage-independent growth (Fig. 5e). Overexpression of wild-type *METTL3*, but not the catalytically dead mutant *METTL3* (D395A), increased the protein and mRNA levels of *NEDD4L* in As-T cells with *FTO* deletion and *METTL3* knockdown (Fig. 5f, g).

In addition to m^6^A^10^, FTO has been shown to demethylate the m^6^Am RNA methylation^44^. To determine whether m^6^Am also has a role in FTO’s function, we assessed the effect of knocking down the m^6^Am methyltransferase PCIF1^45–48^. We found that *PCIF1* knockdown has no effect on the regulation of *NEDD4L* by FTO (Supplementary Fig. 5d–f), consistent with a previous report^49^. Next, we investigated potential m^6^A readers responsible for the regulation of NEDD4L by FTO. Since *FTO* deletion increased the mRNA stability of *NEDD4L*, we reckoned that plausible m^6^A readers could be IGF2BP1-3, a group of m^6^A readers that stabilize m^6^A-modified transcripts^37^. Knockdown of *IGF2BP1*, *IGF2BP2*, or *IGF2BP3* decreased the *NEDD4L* mRNA level (Supplementary Fig. 5g–j). Knockdown of all three *IGF2BPs* (*IGF2BP1-3*) diminished the effect of *FTO* deletion on NEDD4L protein and mRNA levels, and mRNA stability (Fig. 5h–j).CLIP-qPCR analysis showed that IGF2BPs bind with the *NEDD4L* transcript, which was decreased in As-T cells as compared with control cells (Fig. 5k). These findings demonstrate that FTO regulates *NEDD4L* RNA stability through m^6^A mRNA methylation and IGF2BPs.

### As increases the FTO stability through impairing autophagy

**Fig. 6:**
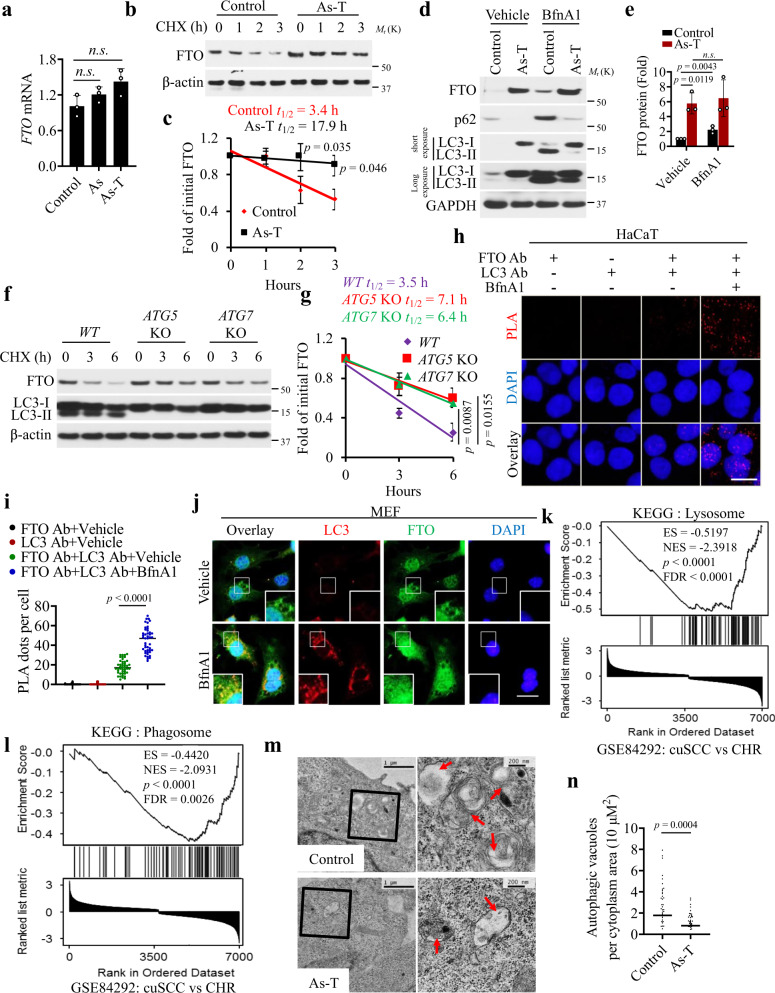
Arsenic impairs autophagic degradation of FTO to stabilize FTO protein. **a** qPCR analysis of *FTO* mRNA levels in control, As, and As-T cells (*n* = 3). **b** Immunoblot analysis of FTO in control and As-T cells treated with cycloheximide (CHX, 100 μg/ml) over a time course. **c** Quantification of **b**. t_1/2_ (half-life) is indicated (*n* = 3). **d** Immunoblot analysis of FTO following treatment with Bafilomycin A1 (BfnA1, 50 nM) for 6 h in control and As-T cells. **e** Quantification of **d** (*n* = 3). **f** Immunoblot analysis of FTO and LC3-I/II in WT, ATG5 KO, and ATG7 KO MEF cells following treatment with CHX (100 μg/ml) over a time course. **g** Quantification of **f**. t_1/2_ (half-life) is indicated (n = 3). **h** Proximity ligation assay (PLA) of the interaction between FTO and LC3 in HaCaT cells treated with vehicle or BfnA1 (50 nM) for 6 h. Scale bar: 20 µm. **i** Quantification of the number of PLA red dots per cell in **h** (*n* = 45 cells). **j** Immunofluorescence analysis of LC3 and FTO in MEF cells treated with vehicle or BfnA1. DAPI is used as a nuclear counterstain (blue). Scale bar:100 µm. **k**, **l** Gene set enrichment analysis (GSEA) of Lysosome (KEGG) (**k**) and Phagosome (KEGG) (**l**) pathway-related genes in mouse UVB-induced cuSCC versus chronically irradiated skin (CHR) in SKH-1 hairless mice. ES: Enrichment score. NES: Normalized enrichment scores, *p*: *p-*value, FDR: False discovery rate. **m**, **n** Electron microscopy (EM) analysis (**m**) and quantification of the number of autophagic vacuoles per cytoplasm area (10 µm^2^) in a cell (*n* = 50 cells) (**n**). Arrows indicate autophagic vacuoles in Control and As-T cells (**m**). Scale bars, 1 µm, and 200 nm. All data were performed on n ≥ 3 biologically independent samples. Error bars are shown as mean ± S.D. (**c**, **e**, **g**, **i**, **n**). *p*-values by two-tailed unpaired *t*-test are indicated (**c**, **e**, **g**, **i**, **n**).

To determine the mechanism by which arsenic upregulates FTO, we first assessed whether arsenic affects *FTO* mRNA levels. As compared with control cells, *FTO* mRNA levels remained unchanged in As and As-T cells (Fig. 6a), indicating that arsenic did not alter *FTO* mRNA abundance. Next, we assessed the effect of arsenic on FTO protein stability by treating the cells with cycloheximide (CHX) over a time course. As compared with control cells, FTO protein stability was increased in As-T cells (Fig. 6b, c), indicating that arsenic increased FTO stability. Next, we assessed the role of protein degradation through either the proteasome or the autophagic-lysosomal pathways, two critical protein degradation pathways^50–52^. When the proteasome was inhibited by MG132, control cells showed FTO protein accumulation, similar to As-T cells (Supplementary Fig. 6a, b). In contrast, when the lysosome was inhibited by the lysosome inhibitor bafilomycin A1 (BfnA1), control cells showed significantly more FTO protein accumulation than As-T cells did (Fig. 6d, e). In addition, As-T cells showed reduced autophagy, as indicated by decreased accumulation of LC3-II, the surrogate marker for autophagosome accumulation, when lysosome was inhibited (Fig. 6d). To further determine the role of autophagy in FTO stability, we assessed the effect of genetic inhibition of autophagy. Deletion of the essential autophagy genes *ATG5* or *ATG7* increased FTO protein stability as indicated by the increased half-life (Fig. 6f, g, and Supplementary Fig. 6c). These findings suggest that arsenic mimics the effect of autophagy inhibition in stabilizing FTO.

Next, we used the PLA assay to assess the plausible interaction between FTO and autophagosomes. We first validated the specificity of the PLA assay for FTO and LC3 (Supplementary Fig. 6d-f). Treatment with BfnA1 increased the interaction between FTO and LC3 (Fig. 6h, i). This was further confirmed by the co-localization of FTO and LC3 by immunofluorescence staining (Fig. 6j). It is noteworthy that FTO was localized mainly in the nucleus, consistent with the previous studies^10^; lysosome inhibition seemed to increase the FTO level in both the nucleus and the cytosol (Fig. 6j). Gene set enrichment analysis (GSEA) detected downregulation of both ‘Lysosome’ and ‘Phagosome’ pathway-related genes in mouse cuSCCs as compared with non-tumor skin (Fig. 6k, l). The downregulated genes were shown in the heatmap (Supplementary Fig. 6g, h). Moreover, we also found that ‘Autophagy-animal’, ‘Lysosome’, and ‘Phagosome’ pathway-related genes are downregulated in As-T cells compared to control cells (Supplementary Fig. 10a, b). Electron microscopy analysis showed that the number of autophagic vacuoles is decreased in As-T cells as compared with control cells (Fig. 6m, n), further supporting the suppression of autophagy by arsenic.

To determine the mechanism by which arsenic inhibits autophagy, we first assessed the role of Metal-activated transcription factor 1 (MTF-1), a multipotent regulator of transcription often involved in the adaptation to stress, including arsenic treatment^53,54^. While low-dose acute or chronic arsenic treatment did not induce MTF1, acute high-dose arsenic treatment did increase the MTF1 level (Supplementary Fig. 6i-l). However, *MTF1* knockdown had no effect on the FTO level (Supplementary Fig. 6m), suggesting that MTF1 is not a major player in chronic low-level arsenic damage. Future studies are required to assess the role of MTF1 in arsenic tumorigenicity in detail. Taken together, these findings demonstrate that arsenic stabilizes FTO protein by inhibiting autophagic degradation.

### p62 regulates FTO stability and is suppressed by arsenic

**Fig. 7:**
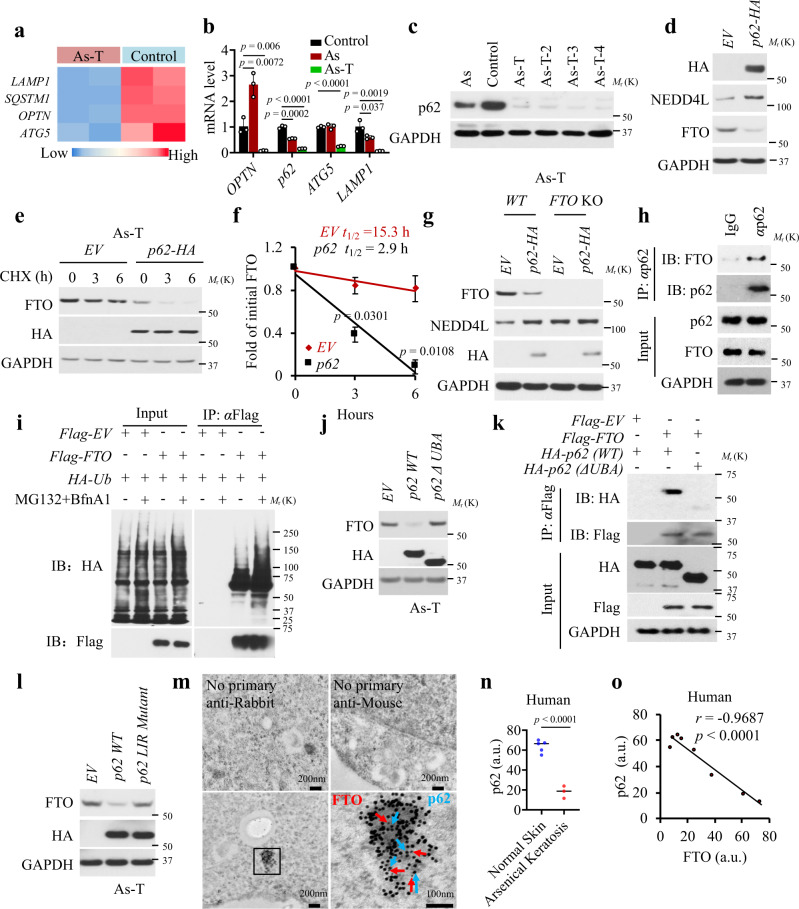
p62 is downregulated by arsenic and regulates FTO stability. **a** Heatmap showing altered expression of the genes in autophagy/lysosome pathways between As-T and control cells. **b** qPCR analysis of *OPTN*, *p62*, *ATG5*, and *LAMP1* levels in control, As, and As-T cells (*n* = 3). **c** Immunoblot analysis of FTO in control, As, and four As-T cells. **d** Immunoblot analysis of HA, NEDD4L, and FTO in As-T cells transfected with *empty vector* (*EV*) or *p62* (*HA* tag). **e** Immunoblot analysis of FTO and HA (p62) in As-T cells as in **d** treated with CHX over a time course. **f** Quantification of **e**. The t_1/2_ (half-life) is indicated (*n* = 3). **g** Immunoblot analysis of FTO and HA (p62) in As-T cells with or without *FTO* deletion in combination with transfection with *EV* or *p62-HA*. **h** Immunoblot analysis of FTO and p62 following immunoprecipitation using species-matched control IgG and an anti-p62 antibody in HaCaT cells. **i** ubiquitination assay of As-T cells transfected with HA-Ub and Flag-FTO and treated with or without BfnA1 (50 nM) and MG132 (10 μM) for 6 h. **j** Immunoblot analysis of FTO and HA in As-T cells transfected with plasmids expressing *WT* and *ΔUBA p62*. **k** Immunoblot analysis of Flag-FTO and HA-p62 (WT or ΔUBA) following immunoprecipitation assay of the binding of Flag-FTO with HA-p62 WT or HA-p62 ΔUBA in HeLa cells transfected with *Flag-EV* (*empty vector*), *Flag-FTO*, *HA-p62 WT*, or *HA-p62 ΔUBA*. **l** Immunoblot analysis of FTO and HA in As-T cells transfected with constructs expressing *WT p62* or *LIR* (*W338A*) *mutant p62* (*HA*). **m** Representative images of immune electron microscopy (IEM) analysis for p62 and FTO in control cells. The upper panel shows the negative controls without primary antibodies. The bottom panel shows IEM staining of FTO (10 nm) and p62 (15 nm). **n** Quantification of the p62 levels in normal human skin (*n* = 6) and arsenical keratoses (*n* = 3). Related to Supplementary Fig. 7j. a.u.: arbitrary units. **o** Negative correlation of p62 levels with FTO levels by Spearman’s test in normal human skin (*n* = 6) and arsenical keratoses (*n* = 3) from Supplementary Fig. 7j. All data were performed on *n* ≥ 3 biologically independent samples. Error bars are shown as mean ± S.D. (**b**, **f**, **n**). *p*-values by two-tailed unpaired *t*-test (**b**, **f**, **n**); Correlation coefficient *r* and *p-*value in **o** are indicated from Spearman’s Correlation Rank test.

To determine the mechanism by which arsenic dysregulates autophagic FTO degradation, we first mined our RNA-seq data for the effect of arsenic on autophagy-related genes. We found that *OPTN*, *LAPM1*, *p62*, and *ATG5* are decreased in As-T cells as compared with control cells (Fig. 7a). qPCR analysis showed that these genes are downregulated in both As and As-T cells as compared to control cells (Fig. 7b). Consistently, the p62 protein level was downregulated by arsenic (Fig. 7c).

Next, we assessed whether p62 regulates FTO protein stability. Indeed, overexpression of p62 reduced the FTO protein level, whereas *p62* knockdown increased it (Fig. 7d and Supplementary Fig. 7a). Moreover, *p62* inhibition increased FTO protein stability in both HaCaT and MEF cells (Supplementary Fig. 7b–e), whereas overexpression of *p62* deceased it in As-T cells that expressed low levels of p62 (Fig. 7e, f). Correspondingly, overexpression of *p62* increased *NEDD4L* mRNA levels in WT cells but not in cells with *FTO* deletion (Fig. 7g and Supplementary Fig. 7f), indicating that p62 regulates *NEDD4L* expression through FTO.

Next, we assessed whether FTO interacts with p62. First, we observed that the co-localization of FTO and p62 is increased by deletion of *ATG7* or by the lysosome inhibitor BfnA1 in MEF cells (Supplementary Fig. 7g). PLA assays supported the co-localization of p62 and FTO in HaCaT cells (Supplementary Fig. 7h, i). Co-immunoprecipitation analysis showed that FTO binds with p62 (Fig. 7h). As an autophagy receptor, p62 can interact with polyubiquitin chains or its ubiquitinated protein substrates through its UBA domain^55^ and with LC3/GABARAP proteins noncovalently via a short linear sequence known as LIR^56^ to deliver its substrates to autophagosomes and then lysosomes for degradation. Indeed, we found that FTO is ubiquitinated in As-T cells (Fig. 7i). Moreover, deletion of the UBA domain of p62 abolished the FTO-p62 interaction (Fig. 7j, k), indicating that FTO-p62 interaction requires p62’s UBA domain. Furthermore, overexpression of wild-type (WT) p62, but not the LIR-binding deficient mutant (W338A)^57,58^, reduced FTO protein levels (Fig. 7l). Immune electron microscopy analysis showed that FTO colocalizes with the p62 cluster in the cytoplasm (Fig. 7m). These p62-positive structures are shown to be liquid droplets formed by liquid-liquid phase separation and degraded by autophagy, rather than monomeric or oligomerized p62^59–61^. It is possible that FTO is translocated into the p62 bodies depending on the context and degraded by autophagy. Lastly, as compared with normal human skin, human arsenical keratoses showed decreased p62 levels (Fig. 7n and Supplementary Fig. 7j). In addition, we found that p62 is negatively correlated with FTO (Fig. 7o).

Next, we assessed the role of p62 in FTO localization in the autophagosomes. Overexpression of p62 increased autophagy in p62-low As-T cells (Supplementary Fig. 8a). In addition, it also increased the co-localization of FTO with LC3 (Supplementary Fig. 8b, c) in As-T cells, whereas *p62* deletion decreased it (Supplementary Fig. 8d-h) in MEF cells. Notably, *p62* deletion markedly decreased but did not completely abolish, the FTO-LC3 PLA signal, suggesting that while p62 is a crucial autophagy receptor for FTO colocalization with LC3, other autophagy factors may also play a role. Immunofluorescence analysis further confirmed that p62 deletion reduced the co-localization of FTO with LC3 (Supplementary Fig. 8i). Our data indicate that p62 is necessary and sufficient for FTO localization in the autophagosomes.

Furthermore, we showed that FTO also regulates autophagy in response to arsenic as a positive feedback signaling system. We found that *FTO* deletion in As-T cells increases autophagy, as shown by p62 accumulation and formation of LC3-II upon BfnA1 treatment (Supplementary Fig. 9a). *FTO* deletion decreased *p62* mRNA levels, consistent with decreased p62 protein levels in FTO-depleted cells under homeostatic conditions (Supplementary Fig. 9a, b). In addition, we found that *FTO* deletion increased the phosphorylation of AMPK, decreased phosphorylation of the mTOR substrate p70S6K, and increased protein levels of ATG5 and ATG7, as well as increasing the LC3-II level, under serum starvation conditions that can induce autophagy^62^ (Supplementary Fig. 9c).

Next, we assessed the role of TFEB, a transcription factor that controls transcription of CLEAR and autophagy genes and is regulated by phosphorylation^63–66^. We found that, as compared with control cells, As-T cells showed reduced levels of TFEB protein and phosphorylation levels (S211), but not the TFEB mRNA level (Supplementary Fig. 9d, e). It is possible that chronic arsenic treatment decreases either translation or protein stability of TFEB and that the decreased TFEB phosphorylation is due to the decreased TFEB abundance, which requires further investigation. *FTO* deletion decreased phosphorylation of TFEB at S122 and the expression of *ATG5* and *ATG7* but not *p62* mRNA (Supplementary Fig. 9f-h), possibly due to the reduced mTOR signaling (Supplementary Fig. 9c). These findings suggest that TFEB has a complex role in arsenic damage response and FTO function, which requires further investigation. Our results suggest a positive feedback loop between FTO and autophagy dysregulation, leading to self-sustained maintenance of FTO upregulation in arsenic-induced malignant transformation and tumorigenesis. Taken together, these findings demonstrate that p62 binds with FTO to mediates FTO degradation, and that arsenic inhibits FTO degradation by suppressing *p62* expression.

### Arsenic downregulates p62 expression by inhibiting the TNF/NF-κB pathway

**Fig. 8:**
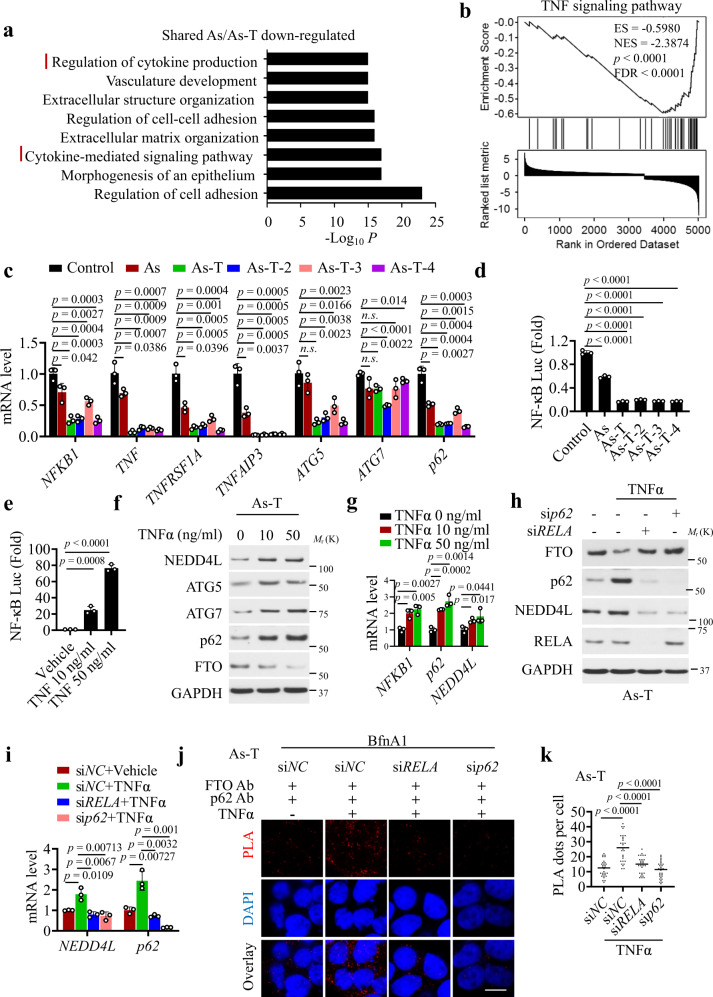
p62 is downregulated by arsenic through TNFɑ/NF-ĸB signaling and regulates FTO stability. **a** GO pathway analysis of downregulated genes shared in both As and As-T cells as compared with control cells. **b** GSEA of “TNF signaling pathway”-related genes in As-T cells versus control cells. ES: Enrichment score. NES: Normalized enrichment scores, *p*: *p*-value, FDR: False discovery rate. **c** qPCR analysis of *NFKB1*, *TNF*, *TNFRSF1A*, *TNFAIP3*, *ATG5*, *ATG7*, and *p62* in control, As, and four As-T cells (*n* = 3). **d** Luciferase reporter analysis of NF-κB activity in control, As, and four As-T cells (*n* = 3). **e** Luciferase reporter analysis of NF-κB activity in As-T cells treated with or without TNFα (10 and 50 ng/ml) for 20 h (*n* = 3). **f** Immunoblot analysis of NEDD4L, ATG5, ATG7, FTO, and p62 in As-T cells treated with or without TNFα for 20 h. **g** qPCR analysis of *NFKB1*, *p62,* and *NEDD4L* mRNA level in cells as in **f** (*n* = 3). **h** Immunoblot analysis of FTO, RELA, p62, and NEDD4L in As-T cells treated with or without TNF (50 ng/ml) following transfection with or without siRNA targeting *p62* or *RELA*. **i** qPCR analysis of p62 and NEDD4L mRNA level in cells as in **h** (*n* = 3). **j** Proximity ligation assay (PLA) of the interaction between FTO and p62 in cells as in **h**. DAPI is used as a nuclear counterstain (blue). All samples are pretreated with BfnA1 for 6 h. Scale bar: 20 µm. **k** Quantification of the number of PLA red dots per cell in **j** (*n* = 30 cells from three biologically independent replicates). All data were performed on n ≥ 3 biologically independent samples. Error bars are shown as mean ± S.D. (**c–e**, **g**, **i**, **k**). *p*-values of all data by two-tailed unpaired t-test are indicated.

To determine how arsenic downregulates *p62* expression, we performed pathway analysis to determine which shared signaling pathways were downregulated in As and As-T cells as compared with control cells using Metascape^67^. Among many pathways downregulated by arsenic, the cytokine production and cytokine signaling pathways, in particular, were downregulated in As and As-T cells as compared with control cells (Fig. 8a). Moreover, KEGG network analysis showed that the ‘Autophagy-animal’,’ Phagosome’, ‘Lysosome’, and ‘NF-κB and TNF signaling pathway’ was downregulated in As-T cells as compared with control cells (Supplementary Fig. 10a, b). GSEA analysis showed that TNF-signaling genes were downregulated in As-T cells as compared with control cells, and in mouse SCC as compared with non-tumor skin (Fig. 8b and Supplementary Fig. 10c-g). qPCR analysis showed that several TNF-related genes (*NFKB1*, *TNF*, *TNFRSF1A*, *TNFAIP3*) and autophagy-related genes (*ATG5*, *ATG7,* and *p62*) were decreased in arsenic-treated cells as compared with control cells (Fig. 8c).

It is known that *p62* expression can be induced by NF-κB activation^68^. Previous studies have shown that in myometrial cells the known NF-κB-inducing cytokine TNFα induces *p62* expression^69^. Therefore, we hypothesized that arsenic downregulates *p62* expression through downregulating signaling of cytokines such as TNFα. Indeed, NF-κB activity was decreased in arsenic-treated cells (As and As-T cells) as compared with control cells (Fig. 8d), while it was increased after TNFα treatment (Fig. 8e). TNFα treatment increased the protein and mRNA levels of *p62* and *NEDD4L*, and the protein levels of ATG5 and ATG7, while it decreased FTO protein level (Fig. 8f, g). Knocking down *p62* or *RELA*, also known as p65 as part of the NF-κB heterodimer, prevented the effect of TNFα (Fig. 8h, i). A PLA assay showed that TNFα increased the colocalization of FTO with either p62 or LC3, which was prevented by knocking down either *p62* or *RELA* (Fig. 8j, k, and Supplementary Fig. 10h, i).

Previous studies showed that Nrf2 and TFEB also regulate *p62* expression^70^. However, we found that in HaCaT cells knockdown of either *Nrf2* or *TFEB* had no effect on FTO levels, although it had a small to moderate effect on the *p62* levels (Supplementary Fig. 10j–l). Future studies are needed to assess the role of Nrf2 and TEFB in arsenic-regulation of p62 in greater detail. Taken together, our findings demonstrate that arsenic suppresses *p62* expression by downregulating the NF-κB pathway to upregulate FTO.

## Discussion

RNA m^6^A modifications are shown to play important roles in cancer development and therapeutic response^7^. However, the role of m^6^A in tumorigenicity caused by environmental carcinogens such as arsenic remains unknown. Here we discovered that chronic low-level arsenic exposure upregulates FTO stability and downregulates m^6^A enrichment in keratinocytes, leading to malignant transformation and tumorigenesis. At the molecular level, arsenic inhibits FTO protein degradation via impairing p62-dependent selective autophagy. FTO interacts with p62 in the autophagosomes, and p62 is required for autophagic degradation of FTO. Furthermore, arsenic inhibits p62 expression by suppressing the TNFα/NF-κB signaling pathway. We identified NEDD4L as a critical downstream target of FTO in arsenic tumorigenicity. *NEDD4L* RNA stability is regulated by m^6^A modification, through the m^6^A readers IGF2BPs and FTO. Furthermore, FTO and autophagy dysfunction form a positive feedback loop to maintain the upregulation of FTO. Taken together, these results establish RNA modification and FTO as an epitranscriptomic mechanism in arsenic-induced tumorigenesis (Supplementary Fig. 11).

We found that FTO is upregulated and m^6^A enrichment is downregulated by chronic exposure to relevant low levels of arsenic. The downregulation of FTO induced by arsenic seems to depend on the dose of arsenic since only low-level arsenic upregulates FTO (Supplementary Fig. 1d). However, high-level arsenic downregulates FTO, similar to previous reports^71,72^. Such FTO upregulation is required for arsenic-induced tumorigenesis, as *FTO* deletion diminished arsenic-induced tumorigenesis in both a xenograft tumor model and an arsenic-UVB co-carcinogenesis model. FTO’s pro-tumorigenic role in arsenic damage response is mediated through m^6^A RNA methylation, as knockdown of *METTL14*/*METTL3* reversed the effect of *FTO* deletion in vivo and in vitro. Furthermore, the FTO level is higher, while the m^6^A level is lower in human arsenical keratosis, a premalignant lesion induced by arsenic than in normal human skin. These findings provide an epitranscriptomic mechanism by which arsenic upregulates the RNA demethylase FTO to promote tumorigenesis.

Emerging evidence suggests that FTO and m^6^A RNA methylation regulate their functions in cancer and other diseases through different downstream gene targets depending on the context and cell lineage^6,7^. For example, in leukemia, Li and colleagues have shown that FTO promotes tumorigenesis by suppressing the expression of *ASB2* and *RARA* genes^11^. In melanoma, we have recently shown that FTO promotes the mRNA stability of the melanoma-promoting genes *PDCD1*, *CXCR4,* and *SOX10*^13^. Using m^6^A IP seq followed by qPCR validation, we found that FTO reduces the m^6^A levels of the *NEDD4L* transcript, leading to its increased decay.

NEDD4L belongs to the NEDD4 family of the E3 HECT domain ubiquitin ligases and has been suggested to regulate several signaling pathways^38,41,42^, thus acting as a tumor suppressor in several cancers^32–35^. We found that knockdown of *NEDD4L* reversed the effect of *FTO* knockout on arsenic-induced tumorigenesis, establishing that *NEDD4L* is a critical mRNA target for FTO. However, in wild-type cells, knockdown of *NEDD4L* had no effect, suggesting that NEDD4L alone is dispensable for arsenic-induced tumorigenicity when FTO is intact to inhibit NEDD4L. In contrast to melanoma in which m^6^A decreases the RNA stability of melanoma-promoting gene transcripts^13^, in arsenic-induced tumor cells, we found that m^6^A increases the RNA stability of *NEDD4L* through the readers IGF2BP1-3. Previously IGF2BP1-3 proteins have been shown to be a distinct family of m^6^A readers that bind to and promote the stability and translation of m^6^A-modified transcripts^37^. We found that knockdown of *IGF2BPs* increases the *NEDD4L* decay in *FTO* knockout cells and reverses the effect of FTO. Future investigations are needed to determine whether IGF2BPs also play active roles in arsenic damage response and tumorigenicity and whether the FTO/IGF2BPs axis also regulates NEDD4L translation.

We found that FTO regulates the Dvl2 level through inhibiting NEDD4L, resulting in increased Wnt signaling, suggesting Wnt signaling as a downstream target for the FTO/NEDD4L axis. Taken together, our findings clearly demonstrate that FTO promotes arsenic-induced tumorigenesis through m^6^A RNA methylation and NEDD4L.

Intriguingly we found that chronic arsenic exposure stabilizes FTO protein by compromising its autophagic degradation. From RNA-seq analysis, arsenic suppressed the expression of several autophagy genes, including the autophagy receptor *p62*. Arsenic inhibited autophagy by suppressing *p62* expression, as adding p62 reversed the effect of arsenic. In addition, we found that FTO degradation by autophagy is mediated by p62. FTO binds with p62’s UBA domain and colocalizes in the LC3-positive autophagosomes in a p62-dependent manner. *p62* knockdown or knockout increases FTO protein stability, while *p62* overexpression decreases it. It appears that arsenic downregulates cytokine signaling pathways, including the TNFα pathway. *p62* expression is shown to be regulated by several signaling pathways and transcription factors such as NF-κB^68^, a major downstream pathway for cytokine signaling. Treatment with TNFα increases NF-κB activity, leading to increased p62 expression and decreased FTO levels. In addition, the roles of other autophagy pathways in FTO degradation will require additional investigation.

Our findings showed that chronic low-level arsenic exposure inhibits *p62* expression, which at least partially leads to compromised autophagy and autophagy-mediated FTO degradation, and thus facilitates arsenic tumorigenesis. Other autophagy genes downregulated by arsenic may also play important roles in autophagy inhibition by arsenic, which needs further investigation. Our findings seem to be specific for arsenic, as previous studies have shown that multiple types of tumors exhibit elevated basal autophagy^73,74^, as well as p62 accumulation^70^, to promote tumorigenesis and tumor progression^70,75,76^. In addition, it seems that the effect of toxic metals on autophagy is also dependent on context, including the specific metal, cell type, concentrations of metals, and treatment duration^77^. We found that in HaCaT cells, cadmium has no effect on FTO levels up to 72 h (Supplementary Fig. 1f). These discrepancies could be due to the specific mechanism of action for arsenic-induced tumorigenesis and suggest a context-dependent role for FTO, as well as p62 and autophagy. The regulatory and functional roles of carcinogenic metals in autophagy warrant further investigation, in particular in the context of clinically relevant low-level chronic exposure.

Moreover, we found that FTO inhibits autophagy. *FTO* deletion increased the expression of autophagy genes including *ATG5* and *ATG7*, increased phosphorylation of AMPK, and decreased phosphorylation of the mTOR target p70S6K and TFEB, pathways that regulate autophagy^63,78,79^, and enhanced autophagy. This is consistent with previous reports in which FTO increases activation of the mTORC1 pathway and thus inhibits autophagy^80^. However, our findings are opposite to those reported in preadipocytes, in which *FTO* knockdown decreases the expression of *ATG5* and *ATG7* and reduces autophagy^81^. These discrepancies could be due to the cell-type specific or context-dependent role of FTO in autophagy, which warrants future investigation. Nevertheless, our findings demonstrate that FTO and autophagy inhibition form a positive feedback loop, leading to self-sustained FTO upregulation and malignant traits in arsenic-induced tumorigenic cells.

In summary, we demonstrate that arsenic promotes FTO protein stability by impairing selective autophagy and thus induces malignant transformation and tumorigenesis. FTO decreases m^6^A modification and stability of its functional downstream target *NEDD4L* mRNA. Our results provide an epitranscriptomic molecular axis as the post-transcriptional mechanism in arsenic-induced tumorigenesis, and thus may help the development of new mechanism-based prevention and therapy by targeting FTO and m^6^A RNA methylation to reduce the skin cancer burden in arsenic-exposed individuals worldwide.

## Methods

### Cell culture

HeLa cells, MEF (mouse embryonic fibroblasts), HaCaT (human keratinocyte, kindly provided by Dr. Fusenig), and HEK-293T cells were maintained in Dulbecco’s modified Eagle’s medium (Invitrogen, Carlsbad, CA) supplemented with 10% fetal bovine serum (Gibco), 100U/ml penicillin, and 100 µg/ml streptomycin (Invitrogen, Carlsbad, CA). Normal Human Epidermal Keratinocyte (NHEK) cells were purchased from Lonza and maintained in KGM Gold keratinocyte growth basal medium (Lonza, #00192151) and KGM Gold keratinocyte growth medium supplements and growth factors (Lonza, # 00192152). WT and ATG5 KO MEF cells were kindly provided by Dr. Noboru Mizushima. ATG7 KO and p62 KO MEF cells were generously provided by Dr. Masaaki Komatsu.

### Lentiviral generation and infection

Lentivirus was produced by co-transfection into HEK-293T (human embryonic kidney) cells with lentiviral constructs together with the pCMVdelta8.2 packaging plasmid and pVSV-G envelope plasmid using X-tremeGENE 9. Virus-containing supernatants were collected at 24–48 h. Target cells were infected in the presence of Polybrene (8 µg/ml) (Sigma-Aldrich, St. Louis, MO) and selected with puromycin (Santa Cruz Biotechnology, Santa Cruz, CA) at 1 µg/ml for 7 days^82,83^.

### Plasmids

Lentiviral vectors expressing shRNA targeting negative control (sh*NC*), *METTL14* (sh*METTL14*), *METTL3* (sh*METTL3*), *FTO* (sh*FTO*), *NEDD4L* (sh*NEDD4L*), *PCIF1* (sh*PCIF1*), and *p62* (sh*p62*) for humans were purchased from Sigma. Plasmids used were as follows: GFP empty vector and *FTO-GFP* (Genecopea). *Flag-EV* and *FLAG-FTO* plasmids were purchased from SinoBiological. pcDNA4-HA-p62 was obtained from Addgene (#28027, kindly provided by Qing Zhong)^84^. p62 with deletion of UBA domain (p62 ΔUBA) was generated as described previously^82^, pLenti-*HA-Ub* purchased from Addgene (#74218, kindly provided by Melina Fan). *p62 LIR-W338A* was subcloned into the pcDNA4 vector (Addgene plasmid #28027, kindly provided by Qing Zhong), from the vector expressing the LIR mutant (pBABEpuro-*HA-p62-LIR*, Addgene plasmid #71306, kindly provided by Jayanta Debnath) using the following primers: Forward, 5‘-CCGGAATTCTATGGCGTCGCTCACCGTGAAGG-3‘; Reverse, 5‘-ATAAGAATGCGGCCGCGCAACGGCGGGGGATGCTTTGAATACTG-3‘). The PCR product was digested with EcoRI and NotI. WT and FTO mutant 1 (H231A/D233A) and mutant 2 (R316Q/R322Q) were generated as described previously^10^. The transcription factor TCF/lymphoid enhancer factor luciferase reporter containing TCF binding sites TOPFLASH (TOP) and its negative control plasmid containing inactive TCF binding sites FOPFLASH (FOP) plasmids were kindly provided by Tong-Chuan He (University of Chicago). pGL4 NF-κB-Luc was obtained from Promega (E8491). *METTL3 WT* and *METTL3 D395A* were provided by Hui-Lung Sun (University of Chicago)^85^. Cells with *FTO* using the CRISPR method were prepared as adapted from the method described previously^8,86,87^. The Cas9 lentiviral vector was kindly provided by Drs. Jiping Xie and Xiaoyang Wu (University of Chicago). The guide RNA sequences were *FTO* KO-1: GAAGCGCACCCCGACTGCCG; *FTO* KO-2: ACGGTCCCCTGGCCAGTGAA. The Tet-On inducible FTO knockdown vectors were kindly provided by Dr. Hui-Lung Sun (University of Chicago), generated by cloning two independent shRNAs targeting FTO: sh*FTO*-1 (shRNA TRCN0000246249 CGGTTCACAACCTCGGTTTAG) and sh*FTO*-2 (shRNA TRCN0000255404, TCTCGCATCCTCATTGGTAAT) into a Tet-On doxycycline-inducible lentiviral expression vector (Addgene Plasmid #21915, kindly provided by Dmitri Wiederschain).

### siRNA transfection

Cells were transfected with siRNA targeting negative control (si*NC*) (Dharmacon, Lafayette, CO), or an individual gene or the combination of *IGF2BP1*, *IGF2BP2*, *IGF2BP3*, *MTF1*, *TFEB*, *Nrf2* using GenMute™ siRNA Transfection Reagent (Signagen, Ijamsville, MD) according to the manufacturer’s instructions^83^.

### Quantitative real-time PCR (qPCR)

Quantitative real-time PCR assays were performed using a CFX Connect real-time system (Bio-Rad, Hercules, CA) with Bio-Rad iQ SYBR Green Supermix (Bio-Rad, Hercules, CA)^83^. The threshold cycle number (CQ) for each sample was determined in triplicate. The CQ values for *FTO*, *NEDD4L*, *ATG5*, *OPTN*, *p62*, *LAMP1*, *IGF2BP1*, *IGF2BP2*, *IGF2BP3*, *ATG7*, *NFKB1*, *TNF*, *TNFRSF1A,* and *TNFAIP3* were normalized against *GAPDH*^83^. Primers are shown in Supplementary Table 1.

### Luciferase reporter assay

Cells were transfected with 1 µg of TOPFLASH/FOP FLASH or NF-κB Luc constructs in combination with 0.025 ĸg of pRL-TK, a transfection efficiency control (Promega, Madison, WI), using GenJetTM Plus DNA in vitro transfection reagent (Signagen, Ijamsville, MD) according to the manufacturer’s instructions. Luciferase reporter assays (Promega) were carried out according to the manufacturer’s instructions^82,83,88^.

### m^6^A dot blot assay

Total RNA was isolated with RNeasy plus Mini Kit (QIAGEN, Hilden, Germany) according to the manufacturer’s instructions. RNA samples were loaded onto the Amersham Hybond-N + membrane (GE Healthcare, Chicago, IL) and UV cross-linked twice to the membrane. After it was dry, the membrane was blocked with 5% BSA (in 1× PBST) for 1 h and incubated with a specific anti-m^6^A antibody (Synaptic Systems, Goettingen, Germany) overnight at 4 °C. Next, the membrane was incubated with the HRP-conjugated anti-rabbit IgG (Cell Signaling Technology, Beverly, MA) for 1 h at room temperature, and then developed with Thermo ECL SuperSignal Western Blotting Detection Reagent (Thermo Fisher Scientific, Waltham, MA)^10,83,89^.

### Gene-specific m^6^A qPCR

Total RNA was immunoprecipitated with an anti-m^6^A antibody followed by qPCR analysis. Primers for full-length mRNA qPCR are listed in the qPCR section. For region-specific m^6^A qPCR of *NEDD4L*, fragmented IP RNA and input RNA samples were prepared as described in the m^6^A IP Seq section and used as RT-qPCR templates for qPCR analysis. Target-specific primers for the m^6^A methylated region in *NEDD4L* (exon 1; NC_000018.10:58221610-58221760) were designed by using Primer-BLAST^90^: forward, 5’-ATCAGCAGAGGTGTGTACGG-3’, and reverse, 5’-CAGTGGTGTCTTTGTTCTTACCT-3’^83,89^.

### CLIP-RT-qPCR

Cells in a 15 cm dish at 70–80% confluency were cross-linked twice with UVC radiation (254 nm, 150 mJ/cm^2^) using Stratalinker on ice. Cells were harvested and lysed, followed by RNase T1 digestion. A 1:1:1 mixture of IGF2BP1-3 antibodies and the corresponding IgG control antibody were conjugated to 1:1 mixed Dynabeads Protein A/G by incubation for 4 h at 4 °C, followed by 3× washing and incubation with pre-cleared cell lysate in RIPA buffer overnight at 4 °C. The beads conjugated with RNA-protein complex were washed and then subjected to digestion by DNase and Proteinase K. Input and co-immunoprecipitated RNAs were recovered by TRIzol (Invitrogen) extraction and used as RT-qPCR templates for qPCR analysis.

### Immunoblotting

Protein extracts were obtained by washing the cells once with PBS and resuspending in RIPA buffer containing inhibitors for proteases and phosphatases, then sonicated. After quantifying protein concentrations through BCA assay, samples were heated for 10 min at 70 °C. Protein abundance was analyzed through SDS–polyacrylamide gel electrophoresis followed by immunoblotting. Antibodies used were as follows: anti-m^6^A (Synaptic system, Cat. # 202 003, 1:2000); anti-FTO (Santa Cruz, SC-271713, 1:500); anti-p62 (Progen Biotechnik GmbH, GP62-C, 1:10,000); anti-METTL14 (Millipore Co., ABE 1338, 1:1000); anti-ATG5 (12994, Cell Signaling Technology, 1:1000); anti-ATG7 (8558, Cell Signaling Technology, 1:1000); anti-GAPDH (Santa Cruz, sc-47724, 1:5000); anti-β-actin (Santa Cruz, SC-47778, 1:5000); anti-ALKBH5 (Millipore Co., ABE 1013, 1:2000); anti-HA (Santa Cruz, sc-7392, 1:5000); anti-IGF2BP1 (Cell Signaling Technology, 8482, 1:1000); anti-IGF2BP2 (Cell Signaling Technology,14672, 1:1000); anti-IGF2BP3 (Cell Signaling Technology, 57145, 1:1000); anti-NEDD4L (Cell Signaling Technology, 4013, 1:1000); anti-LC3 (Abcam, ab192890, 1:1000); anti-p-AMPK T172 (Cell Signaling Technology, 2535S, 1:1000, 1:000), anti-p-ULK1 S555 (Cell Signaling Technology, 5869S, 1:1000); anti-p-p70S6K T389 (Cell Signaling Technology, 9234S, 1:1000); anti-MTF1 (Proteintech, 25383-1-AP, 1:2000); anti-TFEB (Bethyl Laboratories, A303-673A-M, 1:1000); anti-Phospho-TFEB (Ser211) (Cell Signaling Technology, 37681, 1:1000); anti-Nrf2 (Cell Signaling Technology, 12721, 1:1000); Wnt3a (R&D Systems, 5036-WN-010); anti-β-Catenin (Cell Signaling Technology, 9562, 1:5000); Rabbit Anti-Mouse IgG (Cell Signaling Technology, 58802); Mouse Anti-rabbit IgG (Cell Signaling Technology, 5127); HA (Cell Signaling Technology, 3724, 1:2000).

### Immunoprecipitation

Immunoprecipitation was performed using the protocol from Abcam. Briefly, cells were fixed with 0.4% formaldehyde for 10 min and then quenched with 200 mM Tris-Hcl (pH7.5) for 30 min. Fixed cells were scraped with 100 μl of cell harvest buffer, which is 1× cell lysis buffer (10× cell lysis buffer, #9803, Cell Signaling) containing 1% SDS and 5 mM EDTA, and then boiled at 95 °C for 5 min. Next 900 μl of 1× cell lysis buffer (Cell signaling, #9803) was added into the cell lysate in the harvest buffer to quench SDS in the harvest buffer. The cell lysate was mixed well, sonicated, and then incubated with 30 unit/ml of DNase I for 1 h. After centrifuging, the supernatant was used for IP experiments by using Magnetic beads (Protein A/G Magnetic Beads, 88802, Thermo Scientific™), Anti-FLAG® M2 Magnetic Beads (Sigma, M8823, 1:20), HA-Tag (C29F4) (Cell Signaling, #3724, 1:2000), DYKDDDDK Tag (9A3) (Cell Signaling, #8146, 1:2000), METTL3 (Proteintech, 15073-I-AP, 1:1000); Anti-p62/SQSTM1 (Sigma, P0067, 1:100), Anti-FTO (Santacruz, SC-271713, 1:200), Rabbit Anti-Mouse IgG (Light Chain Specific) (D3V2A) (Cell Signaling, #58802, 1:2000), and Mouse Anti-Rabbit IgG (Conformation Specific) (L27A9) (HRP Conjugate) (Cell signaling, #5127 HRP, 1:2000). Immunoprecipitation was performed at 4 °C for 1.5 h and the beads were washed five times in 1× cell lysis buffer (Cell signaling, #9803). Protein was eluted with SDS sample buffer containing Beta-Mercaptoethanol and heated at 95 °C for 20 min^91–94^.

### Ubiquitination assay

The cells were co-transfected with Flag-tagged FTO (Flag-FTO) and HA-tagged ubiquitin (HA-Ub). Then cells were incubated in the presence of MG132 (10 µM) and BfnA1 (50 nM) for 6 h. The ubiquitination assay was performed under denaturing conditions. Briefly, cells scraped with 100 μl of cell harvest buffer, which is 1× cell lysis buffer (10× cell lysis buffer, #9803, Cell Signaling) containing 2% SDS and 5 mM EDTA, and then boiled at 95 °C for 5 min. Next 900 μl of 1× cell lysis buffer (Cell signaling, #9803) was added into the cell lysate in the harvest buffer to quench SDS in the harvest buffer. The cell lysate was mixed well, sonicated. After centrifuging the lysate was subjected to immunoprecipitation by Anti-FLAG® M2 Magnetic Beads (Sigma, M8823, 1:20). Protein was eluted with SDS sample buffer containing DTT and heated at 95 °C for 20 min. All buffers contain protease cocktail inhibitor and 5 mM NEM (N-ethylmaleimide). Ubiquitinated proteins were identified by HA-Tag (C29F4) (Cell Signaling, #3724, 1:2000) antibody^95,96^.

### Electron microscopy (EM)

EM analysis was performed at the electron microscopy core facility on campus. Briefly, cells were fixed and then treated 1% Osmium Tetroxide in 0.1 M sodium cacodylate buffer for 60 min. Then cells were washed with sodium cacodylate buffer, rinsed with Maleate buffer (pH5.1) once for 5 min, stained with 1% aqueous uranyl acetate in Maleate buffer, and dehydrated in a graded series of ethanol. Next cells were embedded by Spurr polymerization in a 60 °C oven. Samples were then sectioned into 90 nm thick sections using Leice EM UC6, stained with Uranyl acetate and Lead citrate, and then examined under 300KV at FEI Tecnai F30.

### Immune electron microscopy (IEM)

IEM analysis was performed at the electron microscopy core facility on campus. Briefly, cells were fixed for 1 h, followed by washing with 0.1 M PB buffer. Samples were then dehydrated with ethanol, embedded, cut into 80 nm-thick slides by Leice EM UC6, and then mounted on formvar/carbon-coated 200 mesh gold grids. Then slides were incubated with antibodies (p62 Sigma-Aldrich 1:20, P0067; FTO abcam, ab92821, 1:20) in 1% BSA. After rehydration with PBS and blocking with 1% BSA, slides were next washed with PBS, followed by blocking with 0.5% BSA again, and then incubated with gold-conjugated secondary antibodies (10 nm for FTO or 15 nm for p62, 1:10) in 0.5% BSA. After washing, samples were stained briefly with Uranyl Acetate and Lead Citrate, air dried, and then examined under 300KV at FEI Tecnai F30.

### Flow cytometric analysis of apoptosis

Apoptotic cell death was determined using an annexin V-FITC apoptosis detection kit (eBioscience, San Diego), according to the manufacturer’s instructions. Cell samples were then analyzed by a BD FACS Calibur flow cytometer (BD Biosciences)^83^.

### Soft agar colony formation

The soft agar assay was performed as described previously^83^. Briefly, cells (500 or 1000 cells) were suspended in 0.35% agar in 1XDMEM/10%FBS growth medium and seeded in 35-mm dishes pre-coated with 0.5% agar in 1× DMEM/10%FBS growth medium, then incubated at 37 °C with 5% CO_2_. Cells were fed 1-2 times per week with a cell culture medium. After 10–14 days, colonies were stained with 0.005% Crystal Violet for more than 1 h.

### Cell proliferation assay

The number of cells was assessed with a Cell Counting Kit-8 (CCK-8) (Sigma-Aldrich, St. Louis, MO following the manufacturer’s protocol^83^.

### Histological analysis

H&E staining was performed by the Immunohistochemistry core facility at the University of Chicago^97,98^.

### Mouse tumorigenesis induced by UVB irradiation and arsenic treatment

All animal procedures have been approved by the University of Chicago Institutional Animal Care and Use Committee. Mice with wild-type (WT; FTO ^*flox/lox*^*, kindly* provided by Dr. Pumin Zhang^99^) and conditional skin-specific *FTO* deletion (*FTO* cKO, *K14Cre*;*FTO* ^*flox/lox*^) in the SKH-1 background were generated^98^. Mice were exposed to arsenite (106277;EMD Millipore) continuously in the drinking water at 1.25 mg/L starting at 21 days of age^27^. Next, at 42 days of age, mice were irradiated with sham or UVB irradiation (starting at 80 mJ/cm^2^ and then increasing the UVB dose by 10% every week until it reached 100 mJ/cm^2^) three times a week up to 25 weeks^97,98,100,101^. Tumor formation and growth were monitored at least weekly until the end of the experiment.

### Tumorigenicity assay in immunocompromised mice

All animal procedures were approved by the University of Chicago institutional animal care and use committee. Athymic nude mice and NOD.Cg-Prkdc^scid^ Il2rg^tm1Wjl^/SzJ (NSG) mice were obtained from Envigo/Charles River and the Jackson Laboratory, respectively. As cells (5 million) in Matrigel or As-T (1 million) cells in PBS with or without gene manipulations were injected subcutaneously into the right flanks of female mice (6–8 weeks of age). For treatment with CS1 or CS2, As-T cells (1 million) in PBS were injected subcutaneously into the right flanks of 6-week-old female nude mice. When tumor size reached about 50 mm^3^, mice were injected i.p. every other day with Vehicle, CS1 (5 mg/kg, Bisantrene, Medchemexpress LLC, Cat. HY-100875), or CS2 (5 mg/kg, Brequinar, Cayman, Cat. 24445) in PBS containing 3% DMSO and 20% HPCD (Sigma-Aldrich, C0926) (total of 8 times). For doxycycline treatment, As-T cells (5 million) expressing inducible FTO knockdown vectors in PBS were injected subcutaneously into the right flanks of 6-week-old female \ nude mice. When tumor size reached about 50 mm^3^, mice were treated with vehicle or 1 mg/ml Doxycycline (MP Biomedicals, Cat. ICN19895501) in acidified drinking water containing 5% sucrose (Sigma, S3929). Tumor growth was monitored and measured by a caliper, and tumor volume was calculated using the formula: tumor volume (mm^3^) = d^2^ × D/2, where d and D are the shortest and the longest diameters, respectively.

### Sphere formation assay

Cells were first harvested in 10% FBS medium and passed through a 40 μm nylon mesh (Fisher, 22363547). Then cells were gently resuspended in a sphere medium (1–2 ml). Cells were next plated in 6-well ultra-low-attachment plates (Corning, 3471) at a density of 1,000 cells per well in culture medium DMEM/F12 (1:1) (Invitrogen) with 2% B27 serum-free supplement (17504-044, Invitrogen, Frederick, MD), 20 ng/ml EGF (E9644, Sigma, St. Louis), 0.4% bovine serum albumin (B4287, Sigma), 4 μg/ml insulin (Sigma, St. Louis, MO, #19278), and antibiotics, and cultured for 7–14 days at 37 °C with 5% CO_2_. The number of tumorspheres per well was quantified^102,103^.

### Immunofluorescence

Cells were first fixed with 4% paraformaldehyde/PBS for 25 min and permeabilized in 0.5% (v/v) Triton X-100 in PBS for 20 min at 4 °C. Then cells were washed with PBS. PBS supplemented with 5% normal goat serum (Invitrogen, Carlsbad, California) was used as a blocking solution for 60 min at room temperature. After removal of the blocking solution, cells were incubated at 4 °C overnight with the following primary antibodies prepared in 1× PBS/1%BSA: anti-LC3B (Abcam, ab192890, 1:200), anti-FTO (Abcam, ab92821, 1:200), and anti-p62 (Sigma-Aldrich, P0067, 1:400). Next cells were washed with PBS, incubated at room temperature in the dark for 1 h with fluorochrome-conjugated secondary antibody (Jackson ImmunoResearch Lab, 715-545-150; Jackson ImmunoResearch Lab, 711-585-152; 1:200) prepared in 1× PBS/1%BSA, followed by washing with PBS three times in the dark. Cells were then fixed in Prolong Gold Antifade with DAPI (Invitrogen) to visualize the cell nuclei and observed under a fluorescence microscope (Olympus IX71)^82,83^.

For tissue IF staining, formalin-fixed, paraffin-embedded tissue sections were pre-treated by antigen retrieval and incubated with blocking solution with 3% albumin from chicken egg white (Sigma-Aldrich, A5503) in PBS after antigen retrieval. For RNA-specific m^6^A staining, DNA on the tissue slide was removed by DNase I treatment. Tissue slides were incubated at 4 °C with primary anti-FTO (Abcam, ab92821, 1:100), anti-NEDD4L (Cell signaling, #4013, 1:100), anti-p62 (Sigma, P0067, 1:100), anti-m^6^A (Synaptic Systems 1: 200), and anti-cytokeratin (ORIGENE, BP5069, 1:200) antibodies. After removing the primary antibodies, slides were washed with PBS solution with 0.1% Triton X-100 (Sigma-Aldrich, T8787). Slides were then incubated at room temperature with Alexa Fluor 594-conjugated secondary rabbit IgG (Jackson ImmunoResearch, 711–585-152, 1:100), Alexa Fluor 488-conjugated secondary mouse IgG (Jackson ImmunoResearch, 715–545-150, 1:100), or DyLight 405 AffiniPure secondary Guinea Pig IgG (Jackson ImmunoResearch, 706-475-148, 1:200) for 1 h, then washed 3 times with PBS solution with 0.1% TritonX-100. Slides were mounted with Fluoromount Mounting Medium (Sigma-Aldrich, F4680). Stained slide samples were analyzed using a fluorescence microscope (Olympus IX71, Olympus Life Science, Japan). For statistical analysis by ImageJ, five areas of the same size were randomly selected from each sample.

### In situ proximity ligation (PLA) assay

PLA assay was performed according to the manufacturer’s instructions (Sigma). Briefly, cells were first fixed with 4% paraformaldehyde/PBS and then permeabilized in 0.5%(v/v) Triton X-100. After blocking with 5% normal goat serum (Invitrogen, Carlsbad, California), cells were incubated overnight with the following primary antibodies at 4 °C in 1XPBS/1%BSA: anti-p62 (Rabbit) (Sigma-Aldrich, P0067, 1:400); anti-LC3B (rabbit) (Abcam, ab192890,1:200), and anti-FTO (mouse) (Abcam, ab92821, 1:200). In-situ PLA detection was performed using a Duolink Detection Kit (Sigma-Aldrich) with a pair of nucleotide-labeled secondary antibodies. After ligation and amplification of PLA probes, signals were examined using a fluorescence microscope (Olympus IX71).

### m^6^A IP seq

100–150 μg total RNA was extracted from cells using TRIzol following the manufacturer’s protocol. mRNA was purified using a Dynabeads mRNA DIRECT Kit (Thermo Fisher, # 61012). 1 μg mRNA was fragmented to ∼200 nt using a Bioruptor® Pico Sonication System, and 5% of the fragmented mRNA was saved as input. mRNA fragments containing m^6^A were enriched with an EpiMark *N*^6^-Methyladenosine Enrichment Kit (NEB, E1610S) and then extracted using RNA Clean and Concentrator (Zymo Research). RNA libraries were prepared for both input and IP samples using TruSeq® Stranded mRNA Library Prep (Illumina, 20020594) following the manufacturer’s protocol. Sequencing was performed at the University of Chicago Genomics Facility on an Illumina NextSeq 4000 machine in single-read mode with 50 bp per reading at around 25 M to 30 M sequencing depth^83^.

### Sequencing data analysis

The adapters were removed by using cutadapt for m^6^A-seq, and reads were aligned to the reference genome (hg38) in Tophat v2.0.14 using the parameter -g 1–library-type = fr-firststrand. RefSeq Gene structure annotations were obtained from the UCSC Table Browser. If a gene had multiple isoforms, the longest isoform was used. In order to eliminate the interference caused by introns in peak calling, aligned reads were extended to 150 bp (average fragment size) and converted from genome-based coordinates to isoform-based coordinates. The method used for peak calling was adapted from published work with modifications^29^. For calling m^6^A peaks, a gene’s longest isoform was scanned using a sliding window (100 bp) with a step of 10 bp. To minimize bias from potential inaccuracy in gene structure annotation and/or the longest isoform, windows with read counts of less than 1/20 of the top window in both m^6^A-IP and the input were excluded. The read counts in each window for each gene were normalized by the median count of all windows for the gene. The differential windows between IP and input samples were identified using a Fisher exact test. If the FDR < 0.01 and log2(Enrichment Score) ≥ 1, the window was considered positive. Overlapping positive windows were merged. To obtain the enrichment score of each peak (or window), the following four numbers were calculated: (1) read counts of the IP samples in the current peak/window, (2) median read counts of the IP sample in all 100 bp windows on the current mRNA, (3) read counts of the input sample in the current peak/window, and (4) median read counts of the input sample in all 100 bp windows on the current mRNA. For each window, the enrichment score was calculated as (a × d)/(b × c)^83^.

### Human skin and tumor samples

All human specimens were studied after approval by the University of Chicago Institutional Review Board. Normal human skin samples were obtained from the archives in the tissue bank of the Section of Dermatology (Department of Medicine, University of Chicago). The arsenical keratoses were obtained from the clinical follow-up for arsenic-exposed cohorts that evaluate the health effects of exposure, including skin cancer, in Bangladesh.

### GEO datasets and analysis of differentially expressed genes (DEG)

Two RNA-seq gene expression profiles (GSE84292, GSE84293)^31^ were acquired from the Gene Expression Omnibus (GEO) database (http://www.ncbi.nlm.nih.gov/geo)^104^, a public functional genomics data repository of high-throughput genomic data. GSE84282 consisted of 6 paired mouse chronically irradiated skin (CHR) and cutaneous squamous cell carcinoma (cuSCC); GSE84283 consisted of 7 cases of human normal skin (NS) and 9 cases of human cuSCC. The DEG was performed by NetworkAnalyst (http://www.networkanalyst.ca), and R programming languages-based tool for comprehensive gene expression profiling^105^. The edgeR method was selected with adjusted *P* < 0.05 to identify the DEGs between mouse and human chronically irradiated skin (CHR) and cuSCC.

### GO, KEGG, GSEA, Venn diagram, and heatmap analysis

To understand the function of the differentially expressed genes, gene ontology (GO) enrichment and Kyoto Encyclopedia of Genes and Genomes (KEGG) pathway enrichment analyses were performed using Metascape and Networkanalyst. *P* < 0.05 was considered to indicate a significantly enriched DEG. Metascape (https://metascape.org/) is a free gene annotation and analysis resource that helps biologists make sense of one or multiple gene lists^67^. Gene Set Enrichment Analysis (GSEA) and the heatmap of relative significant genes were performed using Networkanalyst and WebGestalt (http://www.webgestalt.org/^106^. Venn diagrams were generated using the following web tool: https://bioinfogp.cnb.csic.es/tools/venny/index.html^107^, with the following statistical cutoff: *p-*value <0.05 in combination with log_2_ (fold change) >0.5 or < −0.5 for human cuSCC/CHR and mouse cuSCC/NS (GSE84292, GSE84293), or log_2_ (TPM) > 0.5 or < −0.5 for As-T cells/Control cells.

### Statistical analyses

Statistical analyses were carried out using Prism 7 and 9 (GraphPad). Data were expressed as the mean of at least three independent experiments. Error bars indicate the SDs or SEs of the means. *P* < 0.05 was considered statistically significant.

### Reporting summary

Further information on research design is available in the Nature Research Reporting Summary linked to this article.

## Supplementary information

Supplementary Information

Reporting Summary

## Data Availability

m^6^A IP sequencing and RNA sequencing data are accessible at the GEO repository, under accession number GSE145923. Other data from this study are available from the corresponding author upon request. Source data are provided with the paper. Source data are provided with this paper.

## Source data

Source Data
