# Supplementary material for: Autophagy of the m6A mRNA demethylase FTO is impaired by low-level arsenic exposure to promote tumorigenesis
Source: Nat Commun. 2021 Apr 12;12:2183. doi: 10.1038/s41467-021-22469-6 (PMC8041927; doi:10.1038/s41467-021-22469-6)
Supplement: Supplementary file 1 — Supplementary Information [file 41467_2021_22469_MOESM1_ESM.pdf]

Autophagy of the m6A mRNA demethylase FTO is impaired by low-level arsenic exposure to promote tumorigenesis

Cui et al

Supplemental figures, figure legends, and Table

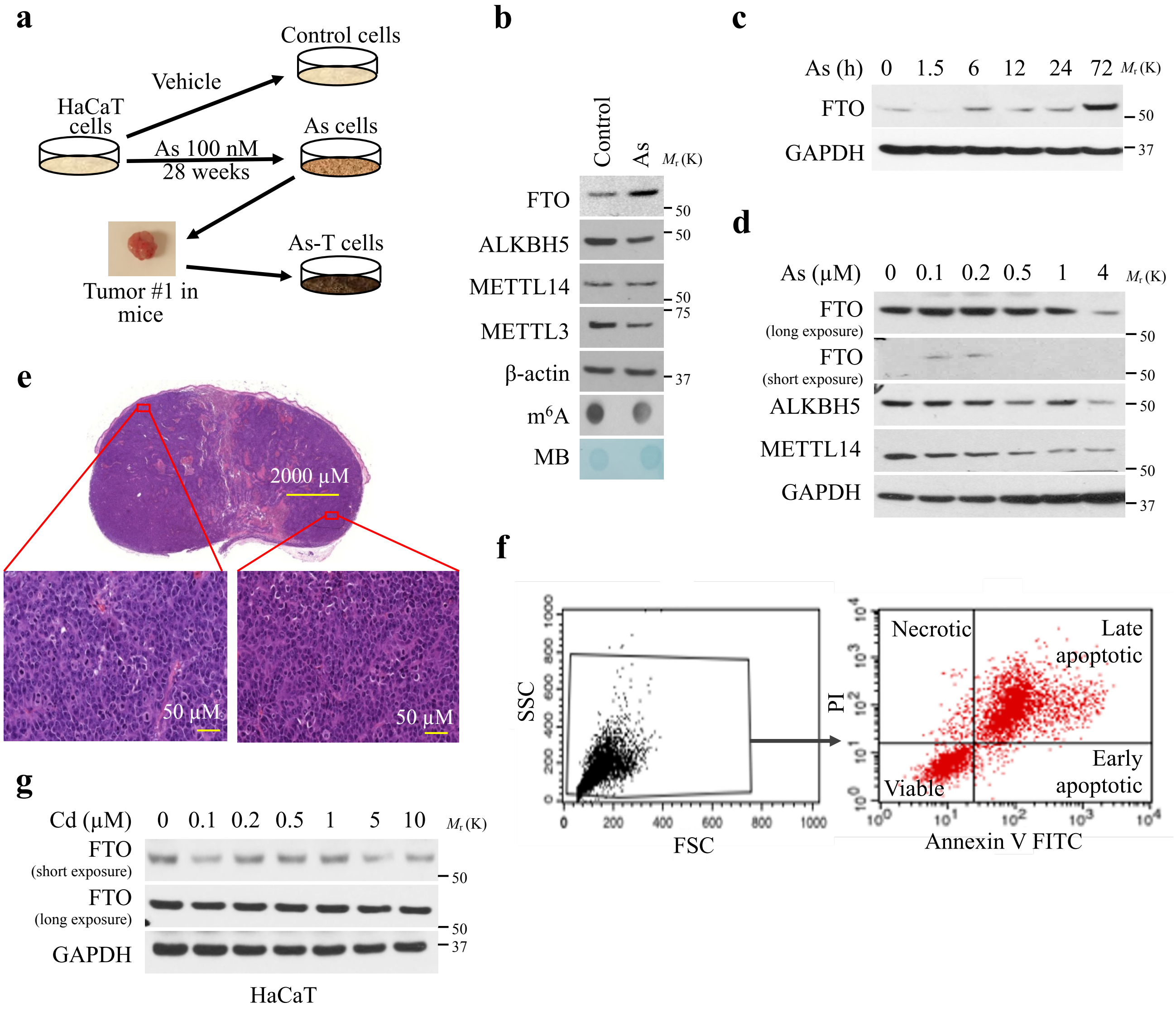

Supplementary Figure 1. related to figure 1 FTO is upregulated in arsenic-treated keratinocytes. a Schematic summary of arsenic experimental design. **b** Immunoblot analysis of the m^{6}A regulators, and dot blot analysis of m^{6}A levels in control and As cells. Methylene blue (MB) staining was used as the loading control. **c** Immunoblot analysis of FTO in HaCaT cells treated with or without arsenic (As, 0.2 μM) over a time course. **d** Immunoblot analysis of FTO in HaCaT cells treated with different doses of arsenic (As) for 24 h. **e** Histological analysis of the As-T tumor at different magnifications (n=4). **f** Gating strategy for Fig. 1f, 4l, and supplementary Fig. 2c. **g** Immunoblot analysis of FTO in HaCaT cells treated with different doses of cadmium (Cd) for 72 h. All data shown are from n ≥ 3 biologically independent samples.

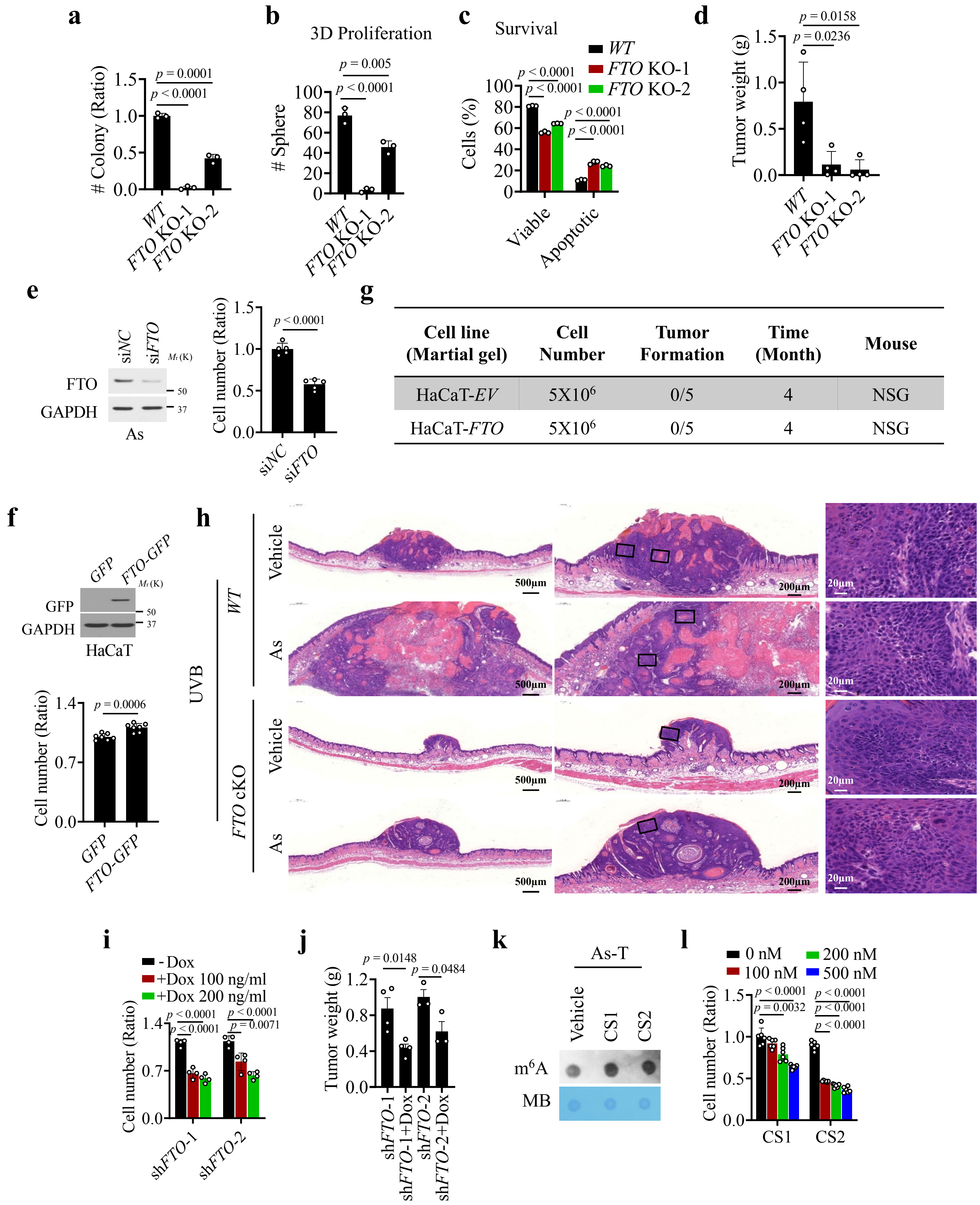

g

Cell line (Martial gel)	Cell Number	Tumor Formation	Time (Month)	Mouse
HaCaT-*EV*	5X10^6	0/5	4	NSG
HaCaT-*FTO*	5X10^6	0/5	4	NSG

Supplementary Figure 2. related to figure 2 FTO is required for arsenic-induced tumorigenicity. **a** Clonogenic assay in As-T cells with or without *FTO* deletion (n=3). **b** Sphere-forming assay of 3D proliferation in cells as in **a** (n=3). **c** Apoptosis assay in cells as in **a** (n=3). **d** Tumor weight from **Fig. 2d**

in As-T cells with or without *FTO* deletion after inoculation in NSG mice (n=4). **e** Cell proliferation assay in As cells with or without *FTO* knockdown (n=3). **f** Cell proliferation assay in HaCaT cells with or without overexpression of *FTO* (n=3). **g** Tumor formation of HaCaT with or without *FTO* overexpression in NSG mice (n=5). **h** Representative images for histological analysis of skin tumors from mice treated with UVB irradiation in combination with vehicle or arsenic. The tumors are either papillomas or squamous cell carcinomas (SCC). Sham group: *WT*+Vehicle (n=6); *WT*+As (n=6); *FTO* cKO+Vehicle (n=5); *FTO* cKO+As (n=6); groups treated with UVB (n=6). **i** Cell proliferation assay of As-T cells with or without inducible *FTO* knockdown (Tet-On sh*FTO*-1 and sh*FTO*-2) treated with or without doxycycline (100 and 200 ng/ml) (n=4). **j** Tumor weight for subcutaneous injection of cells as in **h** in nude mice followed by treatment with or without doxycycline (1 mg/ml Doxycycline). sh*FTO*-1 (n=4) and sh*FTO*-2 (n=3). **k** m^6A dot blot analysis in As-T after treatment with vehicle, CS1 (200 nM) and CS2 (200 nM) for 72 h. **l** Cell proliferation assay in cells as in **j** (n=6). All data were performed on $n \geq 3$ biologically independent samples. Error bars are shown as mean ± S.D. (**a-f**, **i**, **j**, **l**). *p*-values of all data by two-tailed unpaired t-test are indicated.

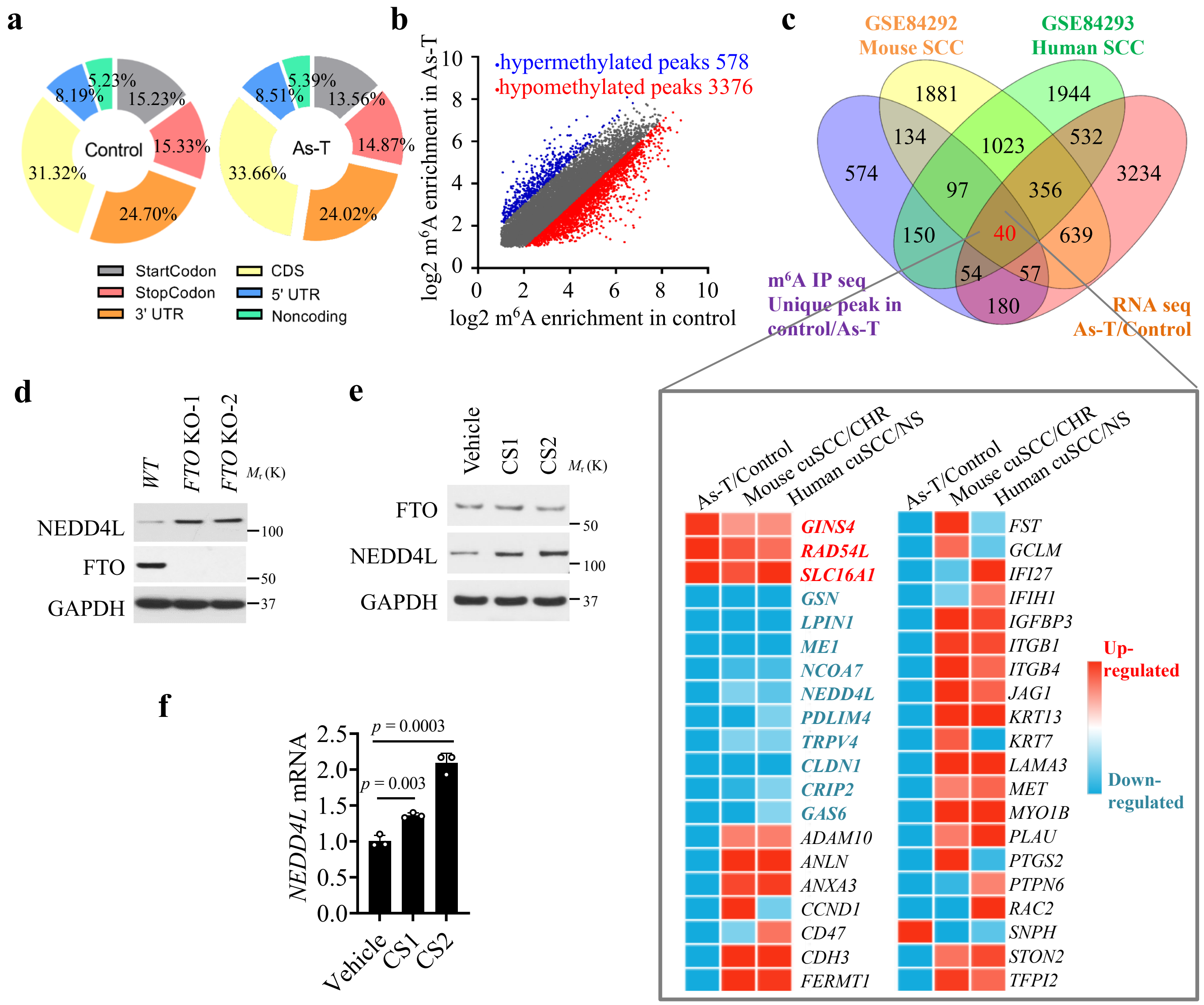

Supplementary Figure 3. related to figure 3 NEDD4L is critical target of FTO. a The proportion of m^{6}A peak distribution in the indicated regions across the entire set of mRNA transcripts in control and As-T cells. **b** Scatter plots showing hypomethylated and hypermethylated genes in As-T cells as compared with control cells. **c** Venn diagram (upper panel) and heatmap (lower panel) showing the overlap between genes with altered expression or m^{6}A enrichment in four datasets: m^{6}A seq (unique m^{6}A peak in control/As-T), RNA seq (As-T/control), RNA seq [Mouse cuSCC/ chronically irradiated skin (CHR)], and RNA seq [human cuSCC/normal skin (NS)]. Heatmap (right) shows normalized value for the 40 genes altered in all four datasets. **d** Immunoblot analysis of NEDD4L and FTO in As-T cells with or without *FTO* deletion. **e** Immunoblot analysis of NEDD4L and FTO in As-T cells treated with vehicle, CS1 (200 nM), and CS2 (200 nM). **f** qPCR analysis of *NEDD4L* mRNA in cells as in **e** (n=3). All data were performed on n ≥3 biologically independent samples. Error bars are shown as mean ± S.D. (**f**). *p*-value by two-tailed unpaired t-test (**f**) is indicated.

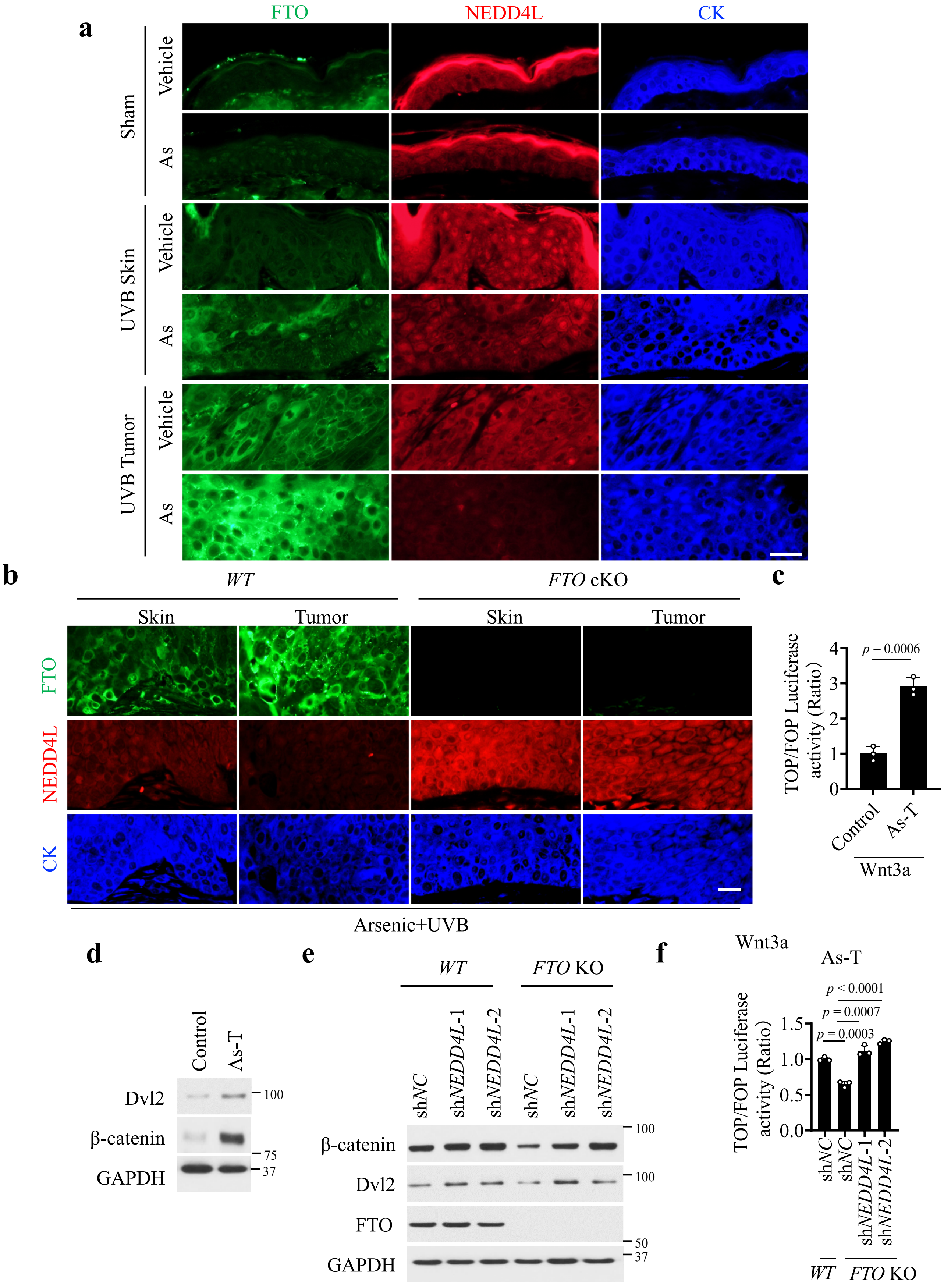
a
FTO
NEDD4L
CK
Sham
Vehicle
As
UVB Skin
Vehicle
As
UVB Tumor
Vehicle
As
b
WT
FTO cKO
Skin
Tumor
Skin
Tumor
FTO
NEDD4L
CK
Arsenic+UVB
c
TOP/FOP Luciferase activity (Ratio)
p = 0.0006
0
1
2
3
4
Control
As-T
Wnt3a
d
Control
As-T
Dvl2
β-catenin
GAPDH
100
75
37
e
WT
FTO KO
shNC
shNEDD4L-1
shNEDD4L-2
shNC
shNEDD4L-1
shNEDD4L-2
β-catenin
Dvl2
FTO
GAPDH
100
100
50
37
f
Wnt3a
As-T
TOP/FOP Luciferase activity (Ratio)
p < 0.0001
p = 0.0007
p = 0.0003
0.0
0.5
1.0
1.5
shNC
shNC
shNEDD4L-1
shNEDD4L-2
WT
FTO KO

Supplementary Figure 4. related to figure 4 NEDD4L is a critical downstream target of FTO in arsenic induced tumor. a Immunofluorescence analysis of FTO (green), NEDD4L (red), and cytokeratin (CK, blue) in non-tumor skin and tumors from female WT mice treated with or without arsenic exposure (n=6). Scale bar: 20 μm. **b** Immunofluorescence analysis of FTO (green), NEDD4L (red), and cytokeratin (CK, blue) in non-tumor skin and tumors in *WT* (n=6) or *FTO* cKO (n=6) female mice treated with both arsenic and UVB exposure. Scale bar: 20 μm. **c** TOP/FOP luciferase reporter assay in control and As-T cells treated with Wnt3a (100 ng/ml) for 24 h (n=3). **d** Immunoblot analysis of Dvl2 and β-catenin in control and As-T cells. **e** Immunoblot analysis of FTO, Dvl2, and β-catenin in As-T cells with or without *FTO* deletion and *NEDD4L* knockdown. **f** TOP/FOP luciferase reporter assay in cells as in **e** but treated with Wnt3a (100 ng/ml) for 24 h (n=3). All data were performed on n ≥3 biologically independent samples. Error bars are shown as mean ± S.D. (**c, f**). *p*-values of all data by two-tailed unpaired t-test are indicated.

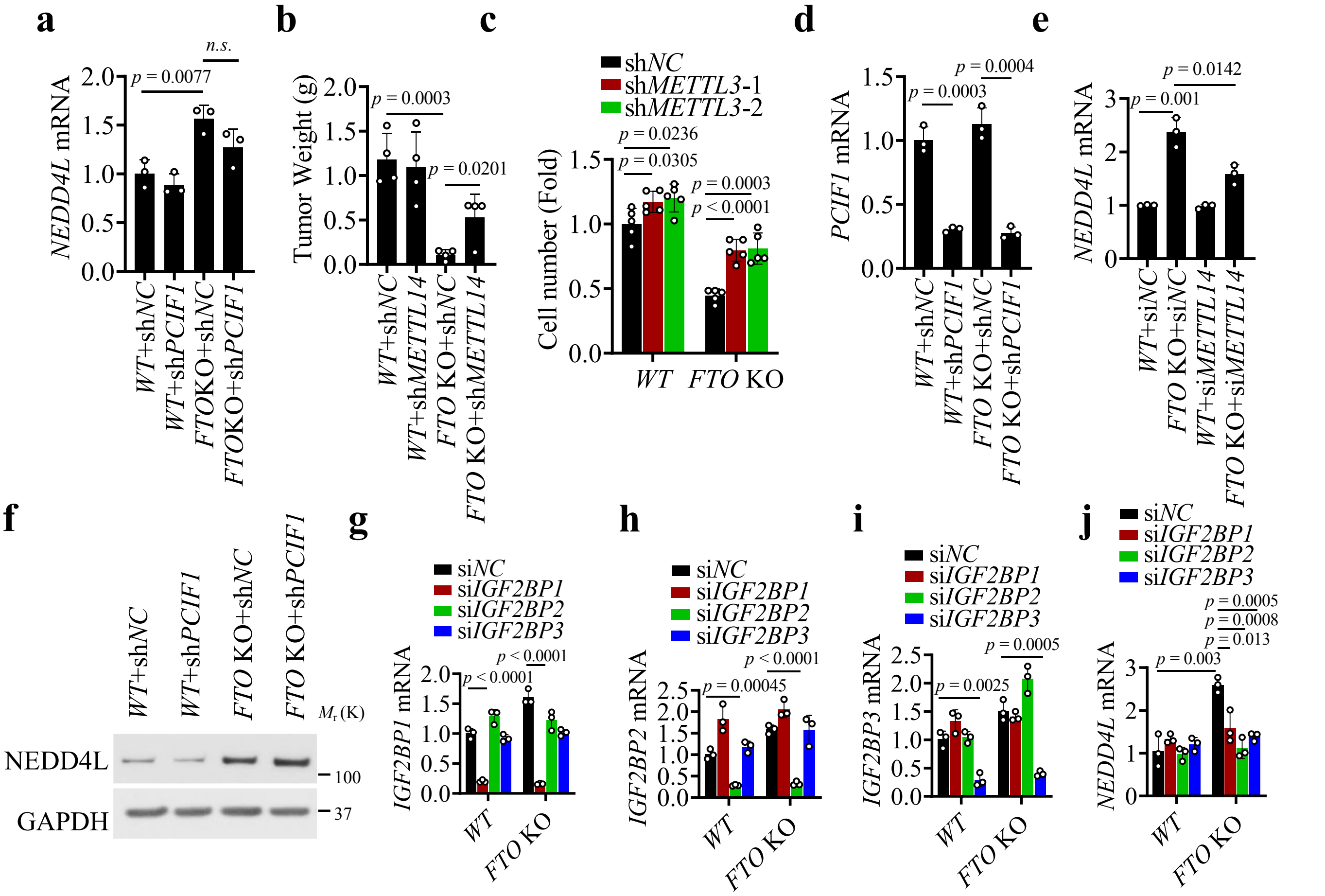

Supplementary Figure 5. related to figure 5 FTO regulates NEDD4L through m^{6}A not m^{6}Am. a qPCR analysis of the mRNA level of *NEDD4L* in As-T cells with or without *FTO* deletion and/or *METTL14* knockdown (n=3). **b** Tumor weight from cells as in **a** following subcutaneous injection into NSG mice (n=4). **c** Cell proliferation assay of As-T cells with or without *FTO* deletion and/or *METTL3* knockdown (n=5). **d, e** qPCR analysis of the mRNA levels of *PCIF1* **(d)** (n=3) and *NEDD4L* **(e)** (n=3) in As-T cells with or without *FTO* deletion and/or *PCIF1* knockdown. **f** Immunoblot analysis of NEDD4L and GAPDH in As-T cells with or without *FTO* deletion and *PCIF1* knockdown. **g-j** qPCR analysis of *IGF2BP1* **(g)** (n=3), *IGF2BP2* **(h)** (n=3), *IGF2BP3* **(i)** (n=3), and *NEDD4L* **(j)** (n=3) in As-T cells with or without *FTO* deletion transfected with si*NC*, si*IGF2BP1*, si*IGF2BP2*, or si*IGF2BP3*. All data were performed on n ≥3 biologically independent samples. Error bars are shown as mean ± S.D. (**a-e**, **g-j**). *p*-values of all data by two-tailed unpaired t-test are indicated.

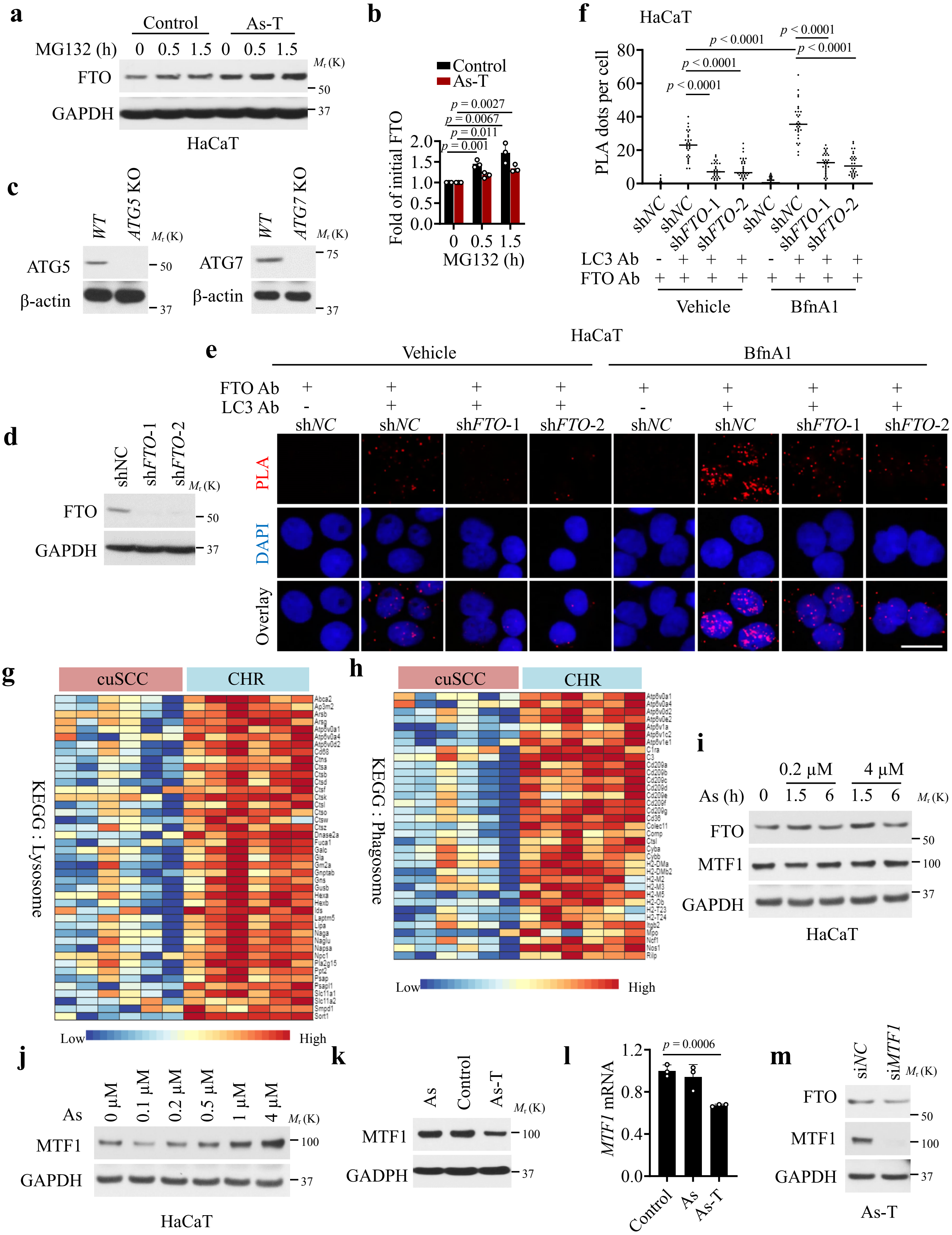

a
Control
As-T
MG132 (h) 0 0.5 1.5 0 0.5 1.5
FTO
GAPDH
M_r (K)
50
37
HaCaT
b
Control
As-T
Fold of initial FTO
p = 0.0027
p = 0.0067
p = 0.011
p = 0.001
0 0.5 1.5
MG132 (h)
c
WT
ATG5 KO
ATG5
β-actin
WT
ATG7 KO
ATG7
β-actin
d
shNC
shFTO-1
shFTO-2
FTO
GAPDH
e
HaCaT
Vehicle
BfnA1
FTO Ab
LC3 Ab
shNC
shFTO-1
shFTO-2
PLA
DAPI
Overlay
f
HaCaT
PLA dots per cell
p < 0.0001
LC3 Ab
FTO Ab
Vehicle
BfnA1
g
cuSCC
CHR
KEGG : Lysosome
Low
High
h
cuSCC
CHR
KEGG : Phagosome
Low
High
i
0.2 μM
4 μM
As (h) 0 1.5 6 1.5 6
FTO
MTF1
GAPDH
HaCaT
j
As
0 μM
0.1 μM
0.2 μM
0.5 μM
1 μM
4 μM
MTF1
GAPDH
HaCaT
k
As
Control
As-T
MTF1
GADPH
l
MTF1 mRNA
p = 0.0006
Control
As
As-T
m
siNC
siMTF1
FTO
MTF1
GAPDH
As-T

Supplementary Figure 6. related to Figure 6 Arsenic impairs autophagic degradation of FTO to stabilize FTO protein. a Immunoblot analysis of FTO in control and As-T cells treated with or without MG132 (10 μM). **b** Quantification of **a**. Average half-life ($t_{1/2}$) is indicated (n=3). **c** Immunoblot analysis of ATG5, ATG7 and β-actin in MEF cells with or without *ATG5* and *ATG7* knockout (KO). **d** Immunoblot analysis of FTO and GAPDH in HaCaT sh*NC*, sh*FTO*-1 and sh*FTO*-2 cells. **e** Proximity ligation assay (PLA) of FTO and LC3 in HaCaT cells with or without FTO knockdown treated with or without Bafilomycin A1 (BfnA1, 50 nM) for 6 h. Cells were counterstained with DAPI (blue). Scale bar: 20 μm. **f** Quantification of the number of PLA red dots per cell in **d**. (n=30 cells from three biological independent replicates). **g,h** Heatmap comparing down-regulated genes in the Lysosome pathway (KEGG) **(f)** and Phagosome pathway (KEGG) **(g)** in mouse cuSCC compared with chronically irradiated skin (CHR). Related to **Fig. 6k** and **6l**, respectively. **i** Immunoblot analysis of FTO and MTF1 in HaCaT cells treated with arsenic (As) at different doses over a time course. **j** Immunoblot analysis of MTF1 in HaCaT cells treated with arsenic (As) at different doses for 72 h. **k** Immunoblot analysis of MTF1 in control, As, and As-T cells. **l** qPCR analysis in cells as in **j** (n=3). **m** Immunoblot analysis of FTO and MTF1 in As-T cells with or without *MTF1* knockdown. All data were performed on n ≥3 biologically independent samples. Error bars are shown as mean ± S.D. (**b**, **f**, **l**). *p*-values of all data by two-tailed unpaired t-test are indicated.

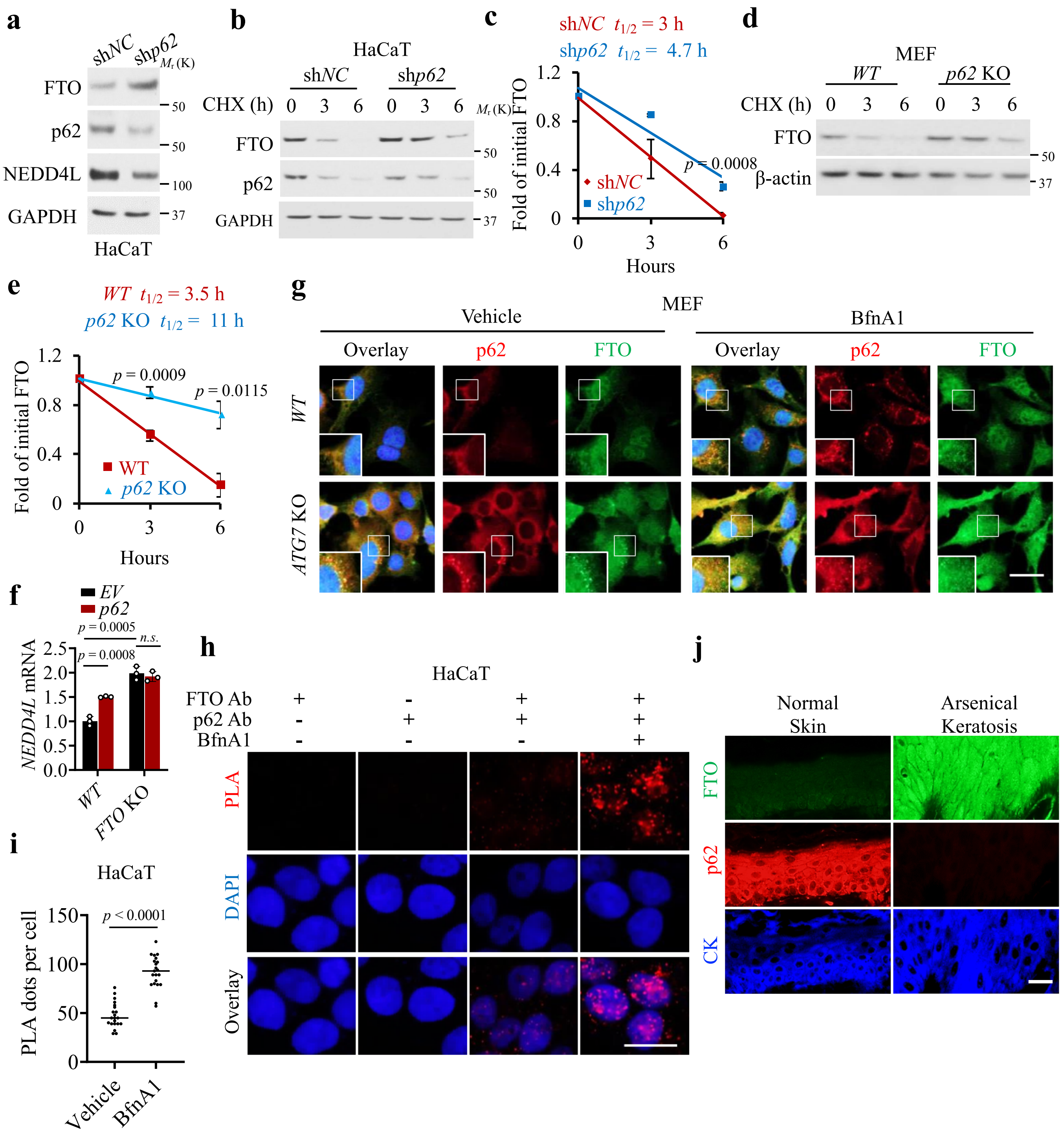

Supplementary Figure 7. related to Figure 7 p62 regulates FTO protein stability. a Immunoblot analysis of FTO and p62 in HaCaT with or without *p62* knockdown. **b** Immunoblot analysis of FTO and p62 in cells as in **a** treated with cycloheximide (CHX, 100 μg/ml) over a time course. **c** Quantification of **b**. Average half-life ($t_{1/2}$) is indicated (n=3). **d** Immunoblot analysis of FTO and β-actin in *WT* and *p62* KO MEF cells with CHX over a time course. **e** Quantification of **d**. Average half-life ($t_{1/2}$) is indicated (n=3). **f** qPCR analysis of *NEDD4L* in As-T cells with or without *FTO* deletion in combination with transfection with *EV* or *p62-HA* (n=3). **g** Immunofluorescence analysis of p62 and FTO in *WT*, *ATG7* KO, and *p62* KO MEF cells with or without BfnA1. DAPI was used as a nuclear counter stain. Scale bar: 100 μm. **h**

Proximity ligation assay (PLA) of FTO and p62 in HaCaT cells treated with vehicle or BfnA1. Scale bar: 20 µm. **i** Quantification of the number of PLA red dots per cell in **h** (n=21 cells from three biological independent replicates). **j** Immunofluorescence staining of FTO (green), p62 (red), and keratin (blue) in normal human skin (n=6) and arsenical keratoses (n=3). Scale bar, 20 µm. All data were performed on n ≥3 biologically independent samples. Error bars are shown as mean ± S.D. (**c**, **e**, **f**, **i**). *p*-values of all data by two-tailed unpaired t-test are indicated.

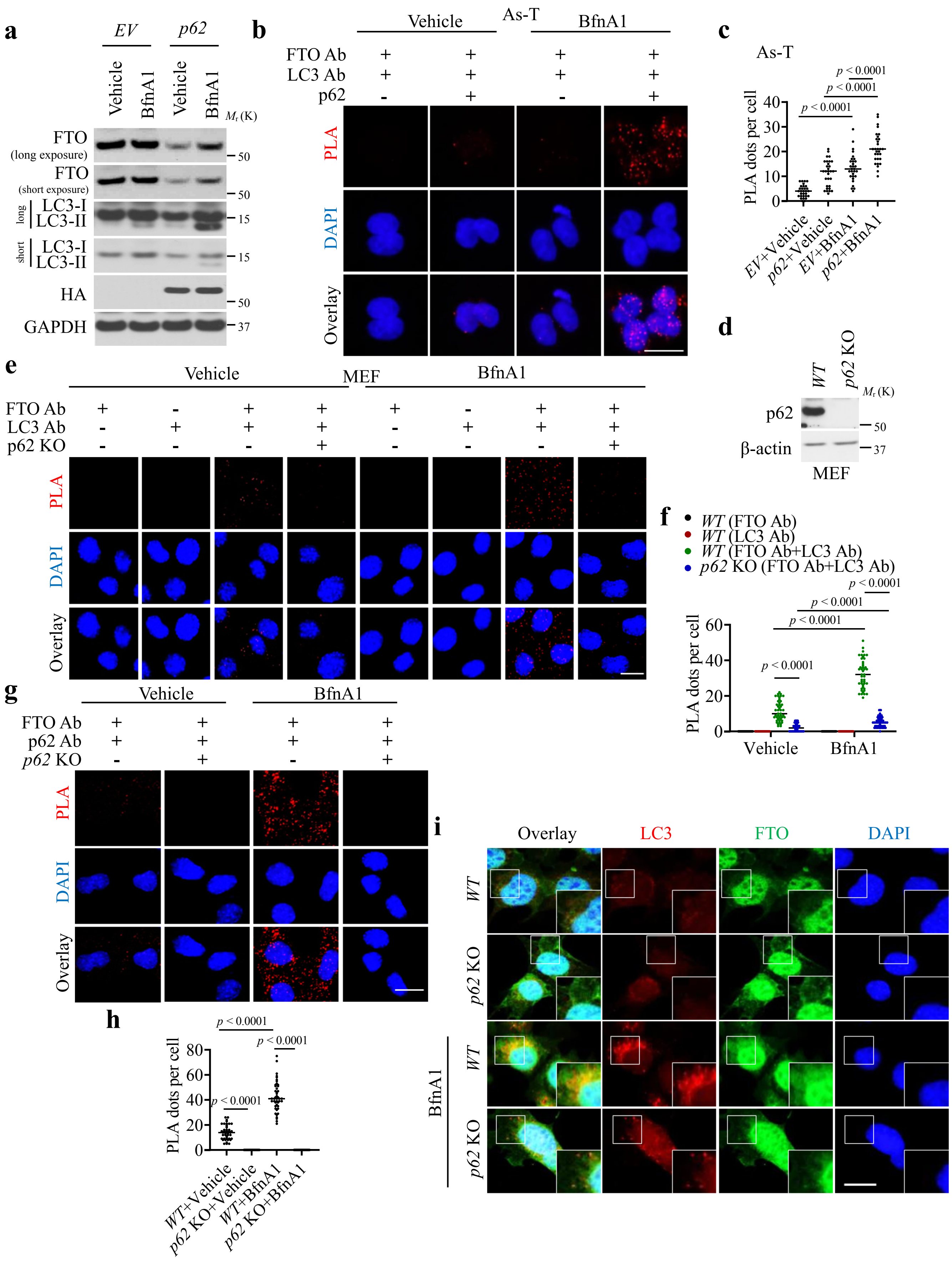

a
EV
p62
Vehicle
BfnA1
Vehicle
BfnA1
M_r (K)
FTO
(long exposure)
FTO
(short exposure)
long
LC3-I
LC3-II
short
LC3-I
LC3-II
HA
GAPDH
50
50
15
15
50
37
b
As-T
Vehicle
BfnA1
FTO Ab
LC3 Ab
p62
PLA
DAPI
Overlay
c
As-T
PLA dots per cell
$p < 0.0001$
$p < 0.0001$
$p < 0.0001$
EV+Vehicle
p62+Vehicle
EV+BfnA1
p62+BfnA1
d
WT
p62 KO
M_r (K)
p62
β-actin
50
37
MEF
e
MEF
Vehicle
BfnA1
FTO Ab
LC3 Ab
p62 KO
PLA
DAPI
Overlay
f
WT (FTO Ab)
WT (LC3 Ab)
WT (FTO Ab+LC3 Ab)
p62 KO (FTO Ab+LC3 Ab)
PLA dots per cell
$p < 0.0001$
$p < 0.0001$
$p < 0.0001$
$p < 0.0001$
Vehicle
BfnA1
g
Vehicle
BfnA1
FTO Ab
p62 Ab
p62 KO
PLA
DAPI
Overlay
h
PLA dots per cell
$p < 0.0001$
$p < 0.0001$
$p < 0.0001$
WT+Vehicle
p62 KO+Vehicle
WT+BfnA1
p62 KO+BfnA1
i
Overlay
LC3
FTO
DAPI
WT
p62 KO
BfnA1
WT
p62 KO

Supplementary Figure 8. related to Figure 7 p62 regulates FTO protein stability through autophagy. a Immunoblot analysis of FTO, LC3-I/II, and HA (p62) in As-T cells transfected with *EV* or *p62* and treated with or without BfnA1 (50 nM) for 6 h. **b** Proximity ligation assay (PLA) of the interaction between FTO and LC3 in cells as in **a**. DAPI is used as a nuclear counterstain (blue). Scale bar: 20 μm. **c** Quantification of the number of PLA red dots per cell in **b** (n=30 cells from three biological independent replicates). **d** Immunoblot analysis of p62 in MEF cells with or without *p62* deletion. **e** Proximity ligation assay (PLA) of FTO and LC3 in MEF cells with or without *p62* deletion treated with vehicle or BfnA1 (50 nM) for 6 h. Scale bar: 20 μm. **f** Quantification of the number of PLA red dots per cell in **e** (n=45 cells from three biological independent replicates). **g** Proximity ligation assay (PLA) of FTO and p62 in *WT* and *p62* KO MEF cells treated with vehicle or BfnA1 (50 nM) for 6 h. Scale bar: 20 μm. **h** Quantification of the number of PLA red dots per cell in **g** (n=45 cells from three biological independent replicates). **i** Immunofluorescence staining of LC3 and FTO in *WT* and *p62* KO MEF cells treated with vehicle or BfnA1 (50 nM) for 6 h. DAPI is used as a nuclear counterstain (blue). Scale bar: 20 μm. All data were performed on n ≥3 biologically independent samples. Error bars are shown as mean ± S.D. (**c**, **f**, **h**). *p*-values of all data by two-tailed unpaired t-test are indicated.

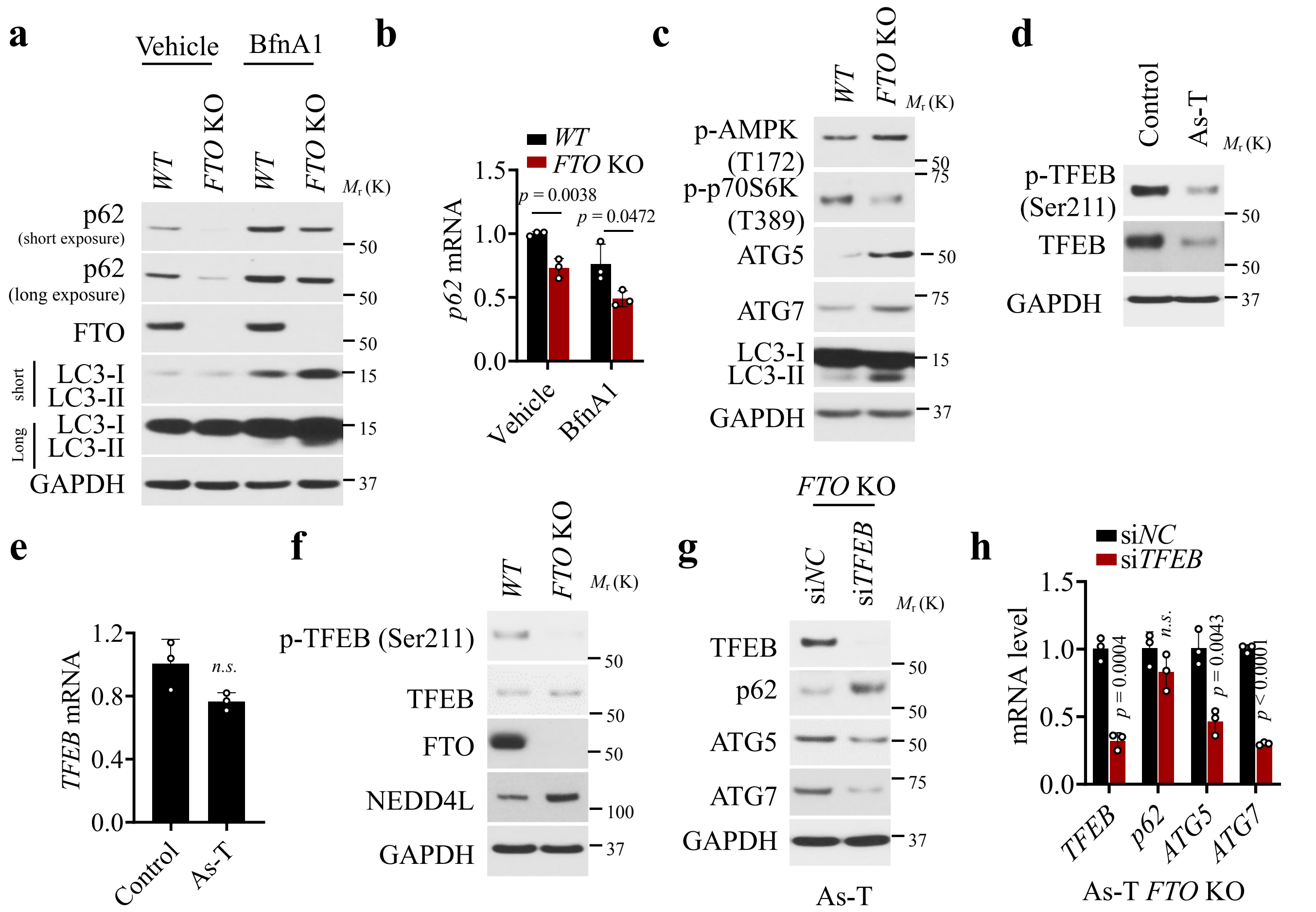

Supplementary Figure 9. related to Figure 7 FTO inhibit autophagy in As-T cells. a Immunoblot analysis of p62, FTO, and LC3-I/II in As-T cells with or without *FTO* deletion and treated with vehicle or BfnA1 (50 nM) for 6 h. **b** qPCR analysis of *p62* mRNA levels in cells as in **a** (n=3). **c** Immunoblot analysis of the autophagy regulators in As-T cells with or without *FTO* deletion and treated with low serum (0.5% FBS) medium overnight. **d** Immunoblot analysis of p-TFEB(Ser211) and TFEB in control and As-T cells. **e** qPCR analysis of *TFEB* mRNA level in control and As-T cells (n=3). **f** Immunoblot analysis of the autophagy regulators in As-T cells with or without *FTO* deletion and treated with low serum (0.5% FBS) medium overnight. **g** Immunoblot analysis of the autophagy regulators in As-T *FTO* KO cells with or without *TFEB* knockdown. **h** qPCR analysis of the mRNA levels of *TFEB*, *p62*, *ATG5* and *ATG7* in cells as in **g** (n=3). All data were performed on n ≥3 biologically independent samples. Error bars are shown as mean ± S.D. (**b**, **e**, **h**). *p*-values of all data by two-tailed unpaired t-test are indicated.

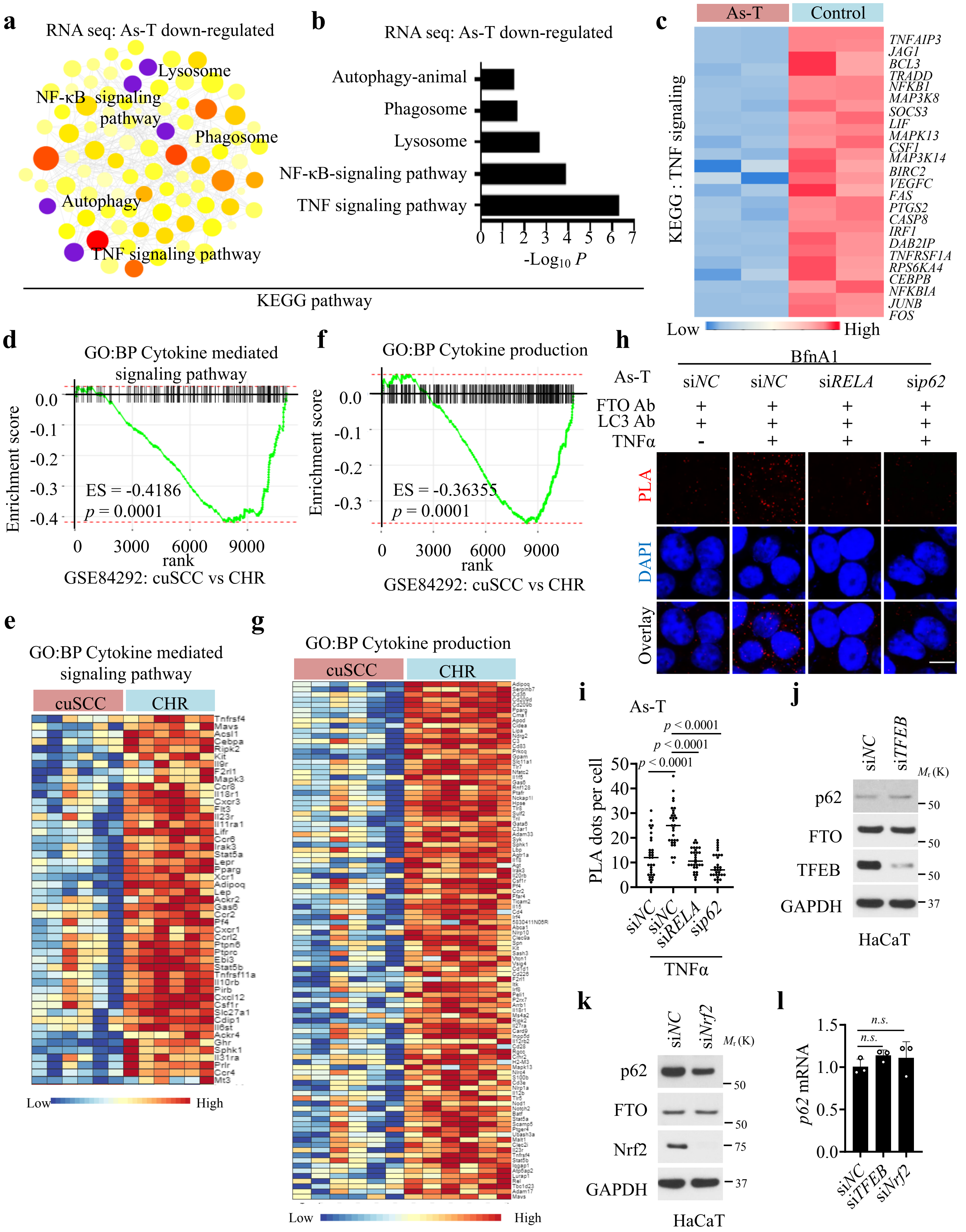
a
RNA seq: As-T down-regulated
Lysosome
NF-κB signaling pathway
Phagosome
Autophagy
TNF signaling pathway
b
RNA seq: As-T down-regulated
Autophagy-animal
Phagosome
Lysosome
NF-κB-signaling pathway
TNF signaling pathway
-Log10 P
KEGG pathway
c
As-T
Control
KEGG : TNF signaling
TNFAIP3
JAG1
BCL3
TRADD
NFKBI
MAP3K8
SOCS3
LIF
MAPK13
CSF1
MAP3K14
BIRC2
VEGFC
FAS
PTGS2
CASP8
IRF1
DAB2IP
TNFRSF1A
RPS6KA4
CEBPB
NFKBIA
JUNB
FOS
Low
High
d
GO:BP Cytokine mediated signaling pathway
Enrichment score
ES = -0.4186
p = 0.0001
rank
GSE84292: cuSCC vs CHR
f
GO:BP Cytokine production
Enrichment score
ES = -0.36355
p = 0.0001
rank
GSE84292: cuSCC vs CHR
h
BfnA1
As-T
siNC
siNC
siRELA
sip62
FTO Ab
LC3 Ab
TNFα
PLA
DAPI
Overlay
e
GO:BP Cytokine mediated signaling pathway
cuSCC
CHR
Low
High
g
GO:BP Cytokine production
cuSCC
CHR
Low
High
i
As-T
p < 0.0001
p < 0.0001
p < 0.0001
PLA dots per cell
siNC
siNC
siRELA
sip62
TNFα
j
siNC
siTFEB
Mr (K)
p62
FTO
TFEB
GAPDH
HaCaT
k
siNC
siNrf2
Mr (K)
p62
FTO
Nrf2
GAPDH
HaCaT
l
n.s.
n.s.
p62 mRNA
siNC
siTFEB
siNrf2

Supplementary Figure 10. related to Figure 8 TNFα signaling regulates FTO stability through NF-κB/p62 signaling. a KEGG network analysis of down-regulated genes in As-T as compared with control cells from RNA seq. Each node represents an enriched KEGG term. **b** Selected pathways in KEGG analysis in **a. c** Heatmap comparing gene expression related to the TNF signaling pathway (KEGG) in As-T and control cells. **d**, **e** GSEA **(d)** and Heatmap **(e)** of down-regulated genes in the Cytokine mediated signaling pathway [Gene Ontology Biological Processes (GO:BP)] in mouse cuSCC as compared with chronically irradiated skin (CHR). ES: enrichment score. *p*: *p*-Value. **f, g** GSEA **(f)** and Heatmap **(g)** of down-regulated genes in the Cytokine production (GO:BP) pathway in mouse cuSCC as compared with chronically irradiated skin (CHR). ES: enrichment score. *p*: *p*-Value. **h** Proximity ligation assay (PLA) of FTO and p62 in As-T cells transfected with or without siNC, sip62 and siRELA, followed by treatment with or without TNFα (50 ng/ml). All samples are pretreated BfnA1 for 6 h. Scale bar: 20 μm. **i** Quantification of the number of PLA red dots per cell in **h** (n=30 cells from three biological independent replicates). **j** Immunoblot analysis of FTO, p62, and TFEB in HaCaT cells with or without *TFEB* knockdown. **k** Immunoblot analysis of FTO, p62, and Nrf2 in HaCaT cells with or without *Nrf2* knockdown. **l** qPCR analysis of the p62 mRNA level in HaCaT cells with or without knockdown of *TFEB* or *Nrf2* (n=3). All data were performed on n ≥3 biologically independent samples. Error bars are shown as mean ± S.D. (**i**, **l**). *p*-values of all data by two-tailed unpaired t-test are indicated.

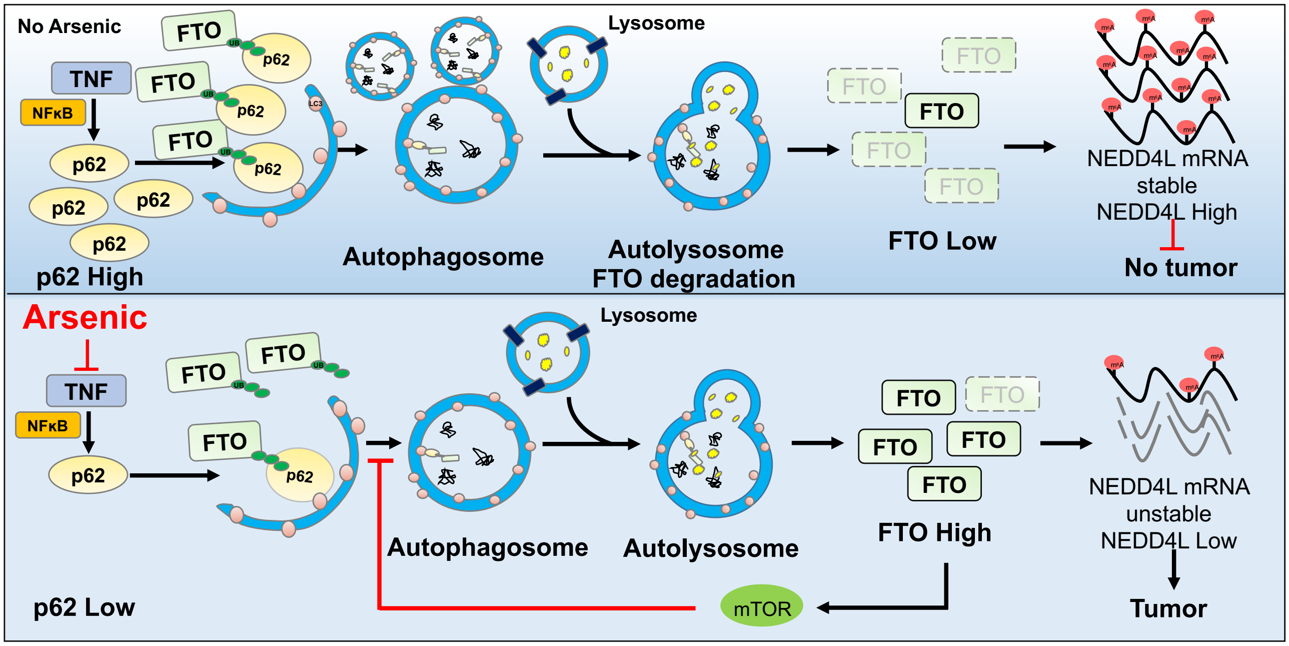

Supplementary Figure 11. related to Figure 1-8 Schematic summary. Schematic summary of proposed model for FTO regulation and function in arsenic-induced tumorigenesis.

Supplementary Table 1: Primers for qPCR analysis

Gene name	Forward	Reverse
IGF2BP1	5`-atccgcaacatcacaaaaca-3`	5`-attatgggccaggatcttcag-3`
IGF2BP2	5`-ccggaaagaaccatcactgt-3`	5`-aagagtgatgatgcgggaac-3`
IGF2BP3	5`-agttgttgtccctcgtgacc-3`	5-gtccactttgcagagccttc-3`
NEDD4L	5`-ggagccagtgatccgtatgt-3`	5`-caggaagtcgtctcgtgtca-3`
HPRT1	5`-tgctgaggatttggaaaggg-3`	5`-acagagggctacaatgtgatg-3`
GAPDH	5`-agggctgcttttaactctggt-3`	5`-ccccacttgattttggaggga-3`
FTO	5`-acttggctcccttatctgacc -3`	5`-tgtgcagtgtgagaaaggctt -3`
OPTN	5`-tgaaagagcagcgagagaga-3`	5`-ggcaggaatgaatcggaata-3`
p62	5`-aatcagcttctggtccatcg-3`	5`-ttcttttccctccgtgctc-3
ATG5	5`-tcagccactgcagaggtgttt-3`	5`-ggctgcagatggacagttgca-3`
LAMP1	5`-tctcagtgaactacgacacca-3`	5`-agtgtatgtcctcttccaaaagc-3`
ATG7	5'-tttgctatcctgccctct -3'	5'-tgcctcctttctggttct -3'
NFKB1	5`-cctggatgactcttgggaaa-3`	5`-tcagccagctgtttcatgtc -3`
TNF	5`-ctcttctgcctgctgcactttg-3`	5`-atgggctacaggcttgtcactc -3
TNFRSF1A	5`-gtgcctaccccagattgaga-3`	5`-tgtcgatttcccacaaacaa-3
TNFAIP3	5`-atgcaccgatacacactgga-3	5`-cacaagcttccggacttctc -3
