# Supplementary material for: Autophagy of the m6A mRNA demethylase FTO is impaired by low-level arsenic exposure to promote tumorigenesis
Source: Nat Commun. 2021 Apr 12;12:2183. doi: 10.1038/s41467-021-22469-6 (PMC8041927; doi:10.1038/s41467-021-22469-6)
Supplement: Supplementary file 2 — Reporting Summary [file 41467_2021_22469_MOESM2_ESM.pdf]

nature research

Corresponding author(s): Yu-Ying He
Last updated by author(s): Mar 3, 2021

Reporting Summary

Nature Research wishes to improve the reproducibility of the work that we publish. This form provides structure for consistency and transparency in reporting. For further information on Nature Research policies, see our Editorial Policies and the Editorial Policy Checklist.

Statistics

For all statistical analyses, confirm that the following items are present in the figure legend, table legend, main text, or Methods section.

n/a	Confirmed	
☐	☒	The exact sample size (*n*) for each experimental group/condition, given as a discrete number and unit of measurement
☐	☒	A statement on whether measurements were taken from distinct samples or whether the same sample was measured repeatedly
☐	☒	The statistical test(s) used AND whether they are one- or two-sided<br>*Only common tests should be described solely by name; describe more complex techniques in the Methods section.*
☒	☐	A description of all covariates tested
☐	☒	A description of any assumptions or corrections, such as tests of normality and adjustment for multiple comparisons
☐	☒	A full description of the statistical parameters including central tendency (e.g. means) or other basic estimates (e.g. regression coefficient) AND variation (e.g. standard deviation) or associated estimates of uncertainty (e.g. confidence intervals)
☐	☒	For null hypothesis testing, the test statistic (e.g. *F*, *t*, *r*) with confidence intervals, effect sizes, degrees of freedom and *P* value noted<br>*Give P values as exact values whenever suitable.*
☒	☐	For Bayesian analysis, information on the choice of priors and Markov chain Monte Carlo settings
☒	☐	For hierarchical and complex designs, identification of the appropriate level for tests and full reporting of outcomes
☒	☐	Estimates of effect sizes (e.g. Cohen's *d*, Pearson's *r*), indicating how they were calculated

Our web collection on statistics for biologists contains articles on many of the points above.

Software and code

Policy information about availability of computer code

Data collection	For Immunofluorescence analysis: Cell Sens standard Ver.1.17<br>For mRNA level analysis : Bio-Rad CFX Manager.
Data analysis	For Statistical analysis: Prism 7 (Ver. 7.0d), and Prism 9 (Ver. 9.0.0), Image J (1.53e; National Institutes of Health)<br>Sequencing analysis: the adapters were removed by using cutadapt for m6A-seq, reads were aligned to the reference genome (hg38) using Tophat v2.0.14 with parameter -g 1 --library-type=fr-firststrand. RefSeq Gene structure annotations were downloaded from UCSC Table Browser.<br>For Flow Cytometry analysis : BD CellQuest (TM)- Ver. 5.2 and FlowJo software (version 10.5.3; FlowJo LLC)<br>For EM and IEM analysis: 300KV at FEI Tecnai F30<br>GEO database analysis: The differentially expressed genes were performed by NetworkAnalyst 3.0 (http://www.networkanalyst.ca).<br>Gene ontology (GO) enrichment and Kyoto Encyclopedia of Genes and Genomes (KEGG) pathway enrichment analyses: Metascape(https://metascape.org/) and Networkanalyst.<br>Gene Set Enrichment Analysis (GSEA): Networkanalyst and WebGestalt (http://www.webgestalt.org/).

For manuscripts utilizing custom algorithms or software that are central to the research but not yet described in published literature, software must be made available to editors and reviewers. We strongly encourage code deposition in a community repository (e.g. GitHub). See the Nature Research guidelines for submitting code & software for further information.

Data

Policy information about availability of data

All manuscripts must include a data availability statement. This statement should provide the following information, where applicable:

- Accession codes, unique identifiers, or web links for publicly available datasets
- A list of figures that have associated raw data
- A description of any restrictions on data availability

The data have been deposited in the GEO repository with the accession numbers GSE145923 and can be accessed at https://www.ncbi.nlm.nih.gov/geo/query/acc.cgi?acc=GSE145923.

Field-specific reporting

Please select the one below that is the best fit for your research. If you are not sure, read the appropriate sections before making your selection.

☒ Life sciences ☐ Behavioural & social sciences ☐ Ecological, evolutionary & environmental sciences

For a reference copy of the document with all sections, see nature.com/documents/nr-reporting-summary-flat.pdf

Life sciences study design

All studies must disclose on these points even when the disclosure is negative.

Sample size	Generally no statistical analysis was performed to predetermine the sample size. Sample size and number of animals are selected based on our previous experiences of carrying out similar experiments and published work (Yang et al. 2019. Nature communication; Qiang et al. 2020. Autophagy; Qiang et al. 2017. Autophagy). The number of independent experiments are indicated in the figure legends of the manuscript. Three to more independent replicates were used to perform statistical analyses.
Data exclusions	No data were excluded from analysis.
Replication	Experiments were independently repeated two to three times. Replications were successful and were described in the method.
Randomization	For the mouse model, animals were randomly assigned into groups receiving UVB/Arsenic treatment. For the xenograft model, animals were randomly assigned into groups receiving various cell line injections. Randomization (formal or otherwise) was not relevant for other data included in the manuscript. We work with cell cultures and compare specific experimental conditions or wild type versus treated cell lines, which cannot be randomized.
Blinding	For immunofluorescence (IF)/PLA experiment, blind staining and blind analysis were carried out. For other experiments, the investigators were not blinded to group allocation during data collection and/or analysis since all data are obtained and presented in an unbiased manner.

Reporting for specific materials, systems and methods

We require information from authors about some types of materials, experimental systems and methods used in many studies. Here, indicate whether each material, system or method listed is relevant to your study. If you are not sure if a list item applies to your research, read the appropriate section before selecting a response.

Materials & experimental systems

n/a	Involved in the study	
☐	☒	Antibodies
☐	☒	Eukaryotic cell lines
☒	☐	Palaeontology and archaeology
☐	☒	Animals and other organisms
☒	☐	Human research participants
☒	☐	Clinical data
☒	☐	Dual use research of concern

Methods

n/a	Involved in the study	
☒	☐	ChIP-seq
☐	☒	Flow cytometry
☒	☐	MRI-based neuroimaging

Antibodies

Antibodies used	anti-m6A Synaptic system Cat. # 202 003 Goettingen, Germany (1:2000 for WB and 1:200 for IP, 1: 200 for IF)<br>anti-cytokeratin (ORIGENE, BP5069, 1:200 for IF);<br>anti-FTO (Santa Cruz, SC-271713, 1:500 for WB); for WB<br>anti-p62 (Progen Biotechnik GmbH, GP62-C, 1:10,000 for WB);<br>anti-METTL14 (Millipore Co., ABE 1338, 1:1000 for WB);<br>METTL3 (Proteintech, 15073-I-AP, 1:1000 for WB);

anti-ATG5 (12994, Cell Signaling Technology, 1:1000 for WB);
anti-ATG7 (8558, Cell Signaling Technology, 1:1000 for WB);
anti-GAPDH (Santa Cruz, sc-47724, 1:5000 for WB);
anti-ALKBH5 (Millipore Co., ABE 1013, 1:2000 for WB);
anti-MTF1 (Proteintech, 25383-1-AP, 1:2000 for WB);
anti-TFEB (Bethyl Laboratories, A303-673A-M, 1:1000 for WB);
anti-Phospho-TFEB (Ser211) (Cell Signaling Technology, 37681, 1:1000 for WB);
anti-Nrf2 (Cell Signaling Technology, 12721, 1:1000 for WB);
anti-β-Catenin (Cell Signaling Technology, 9562, 1:5000 for WB);
anti-HA-Tag (Cell Signaling Technology, 3724, 1:2000, for IP and WB).
anti-HA (Santa Cruz, sc-7392, 1:5000for WB);
anti-IGF2BP1 (Cell Signaling Technology, 8482, 1:1000 for WB);
anti-IGF2BP2 (Cell Signaling Technology,14672, 1:1000 for WB);
anti-IGF2BP3 (Cell Signaling Technology, 57145, 1:1000 for WB);
anti-NEDD4L (Cell Signaling Technology, 4013, 1:1000 for WB, 1:100 for tissue IF);
anti-LC3B (Abcam, ab192890, 1:1000 for WB; 1:200 for PLA; 1:200 for IF);
anti-p-AMPK T172 (Cell Signaling Technology, 2535S, 1:1000, 1:000 for WB),
anti-p-ULK1 S555 (Cell Signaling Technology, 5869S, 1:1000 for WB);
anti-p-p70S6K T389 (Cell Signaling Technology, 9234S, 1:1000 for WB).
anti-p62 (Sigma-Aldrich, P0067, 1:400 for PLA ; 1:400 for IF and 1:20 for IEM, 1:100 for tissue IF);
anti-FTO (Abcam, ab92821, 1:200 for PLA; 1:200 for IF, 1:20 for IEM, 1:100 for tissue IF).
anti-FLAG® M2 Magnetic Beads (Sigma, M8823, 1:20);
anti-DYKDDDDK Tag (9A3) (Cell Signaling, #8146, 1:2000);
Rabbit Anti-Mouse IgG (Light Chain Specific) (D3V2A) (Cell Signaling,#58802, 1:2000);
Mouse Anti-Rabbit IgG (Conformation Specific) (L27A9) (HRP Conjugate) (Cell signaling, #5127 HRP, 1:2000);
Alexa Fluor 488-conjugated secondary mouse IgG, Jackson ImmunoResearch, Cat# 715-545-150, West Grove, PA (1: 100 for IF)
Alexa Fluor 594-conjugated secondary rabbit IgG, Jackson ImmunoResearch, Cat# 711-585-152, West Grove, PA (1: 100 for IF)
DyLight 405 AffiniPure secondary Guinea Pig IgG (Jackson ImmunoResearch, 706-475-148, 1:200)
Wnt3a (R&D Systems, 5036-WN-010);

Validation

The commercial antibodies were validated based on the information of the manufacturers' instructions and additionally the antibodies were validated by the use of negative control and/or positive control (such as knockout, knockdown or overexpression) for FTO, Nrf2, MTF1, TFEB, METTL3, METTL14, p62, IGF2BP1, IGF2BP2, IGF2BP3, NEDD4L, ATG5 and ATG7 antibodies. For details, siRNA Knockdown effect (Nrf2, MTF1, TFEB, IGF2BP1, IGF2BP2, IGF2BP3); shRNA knockdown effect (p62, METTL3, METTL14). knockout effect (FTO, ATG5, ATG7 and p62); overexpression effect (METTL3).

The following antibodies were validated in knockout/knockdown cells: anti-FTO (Santa Cruz, SC-271713); anti-p62 (Progen Biotechnik GmbH, GP62-C);anti-METTL14 (Millipore Co., ABE 1338); anti-ATG5 (12994, Cell Signaling Technology); anti-ATG7 (8558, Cell Signaling Technology); anti-MTF1 (Proteintech, 25383-1-AP);anti-TFEB (Bethyl Laboratories, A303-673A-M); anti-Nrf2 (Cell Signaling Technology, 12721, 1:1000 for WB); anti-IGF2BP1 (Cell Signaling Technology); anti-IGF2BP2 (Cell Signaling Technology); anti-IGF2BP3 (Cell Signaling Technology, 57145); anti-NEDD4L (Cell Signaling Technology, 4013); METTL3 (Proteintech, 15073-I-AP).

The following antibodies were validated in Co-IP experiments:
anti-HA-Tag (Cell Signaling Technology, 3724); anti-DYKDDDDK Tag (9A3) (Cell Signaling, #8146, 1:2000);anti-p62 (Sigma-Aldrich, P0067); anti-FTO (Abcam, ab92821);

The concentration recommended from antibody's date sheet was used for western blot, dot blot and immunostaining.

Eukaryotic cell lines

Policy information about cell lines

Cell line source(s)

Human HaCaT keratinocytes were kindly provided by Prof. N. Fusenig [German Cancer Research Center (DKFZ), Heidelberg, Germany];
HEK-293 T (human embryonic kidney cells, ATCC, CRL-3216);
Normal Human Epidermal Keratinocyte (NHEK) cells were purchased from Lonza;
MEF(mouse embryonic fibroblast), WT and ATG5 KO MEF cells were kindly provided by Dr. Noboru Mizushima;
WT and ATG7 KO and p62 KO MEF cells were generously provided by Dr. Masaaki Komatsu.

Authentication

All cell lines were authenticated according to the ATCC cell line authentication test recommendations, including morphology check by microscope, growth curve analysis, and mycoplasma test.

Mycoplasma contamination

All cell lines were tested to be mycoplasma negative. All lines were routinely tested for mycoplasma contamination.

Commonly misidentified lines
(See ICLAC register)

No commonly misidentified cell lines listed by ICLAC were used.

Animals and other organisms

Policy information about studies involving animals; ARRIVE guidelines recommended for reporting animal research

Laboratory animals

Athymic Nude Female mice were obtained from Envigo and Charles River. Female mice (6-8 weeks of age) were used in subcutaneous injection.
NSG mice Female (severely combined immunodeficient (NOD/SCID) interleukin-2 receptor (IL-2R) gamma chain null) were obtained

from Jackson Laboratory. 6-8-weeks-old female mice were used in subcutaneous injection.
Female and Male Mice with wild type (WT; FTO flox/lox, kindly provided by Dr. Pumin Zhang) and conditional skin-specific FTO deletion (FTO cKO, K14Cre;FTO flox/lox) in the SKH-1 background were generated used for UVB irradiation and arsenic treatment experiments. 21-days-old female and male mice were treated with arsenic.

Wild animals	No wild animal was used in this research.
Field-collected samples	No field-collected samples were used.
Ethics oversight	All animal procedures used were approved by the University of Chicago institutional animal care and use committee

Note that full information on the approval of the study protocol must also be provided in the manuscript.

Flow Cytometry

Plots

Confirm that:

☒ The axis labels state the marker and fluorochrome used (e.g. CD4-FITC).

☒ The axis scales are clearly visible. Include numbers along axes only for bottom left plot of group (a 'group' is an analysis of identical markers).

☒ All plots are contour plots with outliers or pseudocolor plots.

☒ A numerical value for number of cells or percentage (with statistics) is provided.

Methodology

Sample preparation	For apoptosis assay, cells were dissociated in 0.25% trypsin-EDTA (Gibco) at 37ºC for 5 min and the trypsin was inactivated with 10% FBS DMEM. Then cells were stained with the Annexin V-FITC Apoptosis Detection kit (eBioscience, San Diego), according to the manufacturer's instructions., and then analyzed by BD FACSCalibur flow cytometer (BD Biosciences).
Instrument	BD- FACSCalibur flow cytometer (BD Biosciences)
Software	For data collection and analysis, BD CellQuest (TM)- Ver. 5.2 was used.
Cell population abundance	100,000 cells were collected for analysis.
Gating strategy	Gating strategy was performed using positive and negative samples with Annexin V-FITC and PI staining. For Annexin V staining, positive Annexin V was defined for cells incubated with Annexin V-FITC. Negative Annexin V control was defined for cells that are not incubated with Annexin V-FITC (non-stained). Same strategy is used for PI staining. Statistical analysis was performed on apoptotic cells (the double positive population and single Annexin V-FITC) and viable cells (double negative populations for viable cells).

☒ Tick this box to confirm that a figure exemplifying the gating strategy is provided in the Supplementary Information.
